# Force transmission is a master regulator of mechanical cell competition

**DOI:** 10.1038/s41563-025-02150-9

**Published:** 2025-03-14

**Authors:** Andreas Schoenit, Siavash Monfared, Lucas Anger, Carine Rosse, Varun Venkatesh, Lakshmi Balasubramaniam, Elisabetta Marangoni, Philippe Chavrier, René-Marc Mège, Amin Doostmohammadi, Benoit Ladoux

**Affiliations:** 1 Université Paris Cité, CNRS, Institut Jacques Monod, Paris, France; 2 Niels Bohr Institute, University of Copenhagen, Copenhagen, Denmark; 3 Institut Curie, Paris Université Sciences et Lettres, CNRS, Paris, France; 4 Translational Research Department, Institut Curie, PSL Research University, Paris, France; 5 Department of Physics, Friedrich-Alexander-Universität Erlangen-Nürnberg, Erlangen, Germany; 6 Max-Planck-Zentrum für Physik und Medizin and Max Planck Institute for the Science of Light, Erlangen, Germany

**Keywords:** Biophysics, Adherens junctions, Collective cell migration, Tumour heterogeneity, Mechanotransduction

## Abstract

Cell competition is a tissue surveillance mechanism for eliminating unwanted cells, being indispensable in development, infection and tumourigenesis. Although studies have established the role of biochemical mechanisms in this process, due to challenges in measuring forces in these systems, how mechanical forces determine the competition outcome remains unclear. Here we report a form of cell competition that is regulated by differences in force transmission capabilities, selecting for cell types with stronger intercellular adhesion. Direct force measurements in ex vivo tissues and different cell lines reveal that there is an increased mechanical activity at the interface between two competing cell types, which can lead to large stress fluctuations resulting in upward forces and cell elimination. We show how a winning cell type endowed with a stronger intercellular adhesion exhibits higher resistance to elimination and benefiting from efficient force transmission to the neighbouring cells. This cell elimination mechanism could have broad implications for keeping the strong force transmission ability for maintaining tissue boundaries and cell invasion pathology.

## Abstract (web summary)

Differences in force transmission capabilities between competing cells create large stress fluctuation at their interface, resulting in upward forces and cell elimination, which might have implications for tissue homeostasis and tumour cell invasion.

## Main

Cell competition has a vital role in maintaining tissue health, fighting against pathogens and tumourigenesis^1–4^. Despite these widespread and crucial implications, the fundamental principles that govern cell competition remain unclear. The elimination of loser cells can be facilitated by biochemical signals, which lead to cell death and subsequent removal^1,2^, but various studies have also shown that cells can mechanically outcompete each other^5,6^. The prevailing consensus is that winners compress losers, promoting loser cell’s death and removal^3,5,6^. Different strategies such as directed migration^7,8^, crowding^9^, differences in cell growth^10,11^ or homeostatic density^12,13^ enable winning cells to apply pressure or resist^7–13^. However, contradicting outcomes have emerged from studies exploring the change in cell mechanics through modulating the extracellular environment^14–16^ or changing contractility, for example, by overexpressing the oncogene Ras^V12^ in different in vivo and in vitro systems^17–23^. Although cell competition is involved in various biological and pathological processes, a framework that integrates the role of collective mechanical interactions in cell competition is lacking. In particular, if and how cell competition is influenced by the fundamental process of intercellular force transmission is not known. Sensing, transmitting and exerting mechanical forces between cells is mediated in epithelia by the adherens junction protein E-cadherin (E-cad)^24^, which is crucial for an efficient intercellular mechanical coupling^25–30^. Therefore, we conjectured that altering intercellular force transmission by modifying the E-cad adhesion strength could lead to the emergence of cell competition and strongly affect its outcome.

## Force transmission provides a competitive advantage

**Fig. 1:**
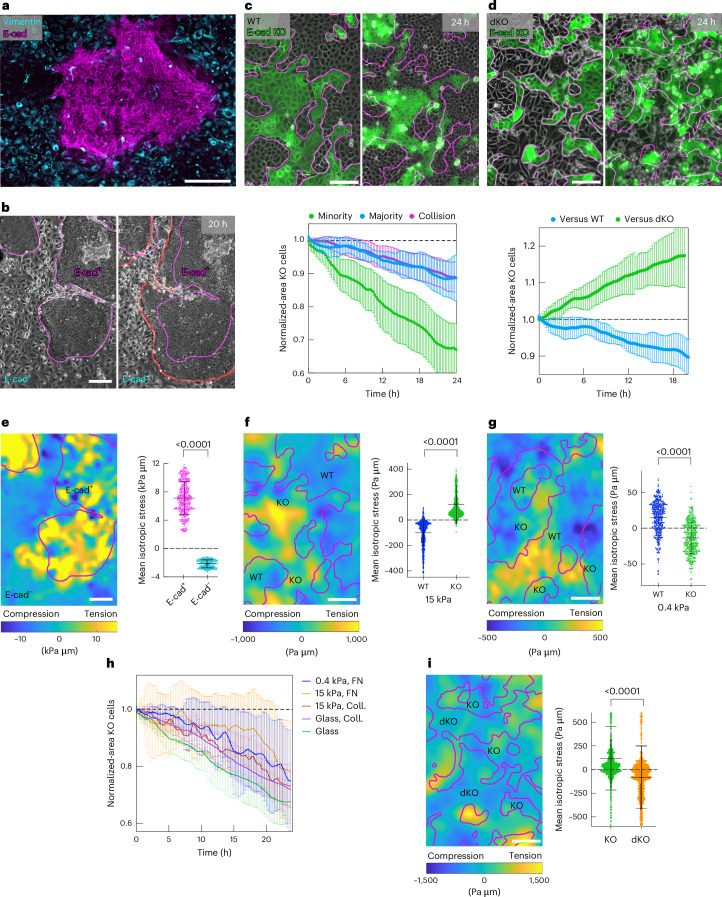
Intercellular force transmission capabilities provide a competitive advantage. **a**, Monolayer of patient-derived metaplastic cancer cells. The E-cad^+^ (magenta) and vimentin-positive (cyan) sub-populations sort completely. **b**, Cluster development. The red line shows cell clusters after 20 h. **c**, Top: mixed culture of MDCK WT (grey) and MDCK E-cad KO (green, fluorescently labelled with LifeAct-GFP) cells. Bottom: area occupied by E-cad KO cells being in minority (green), majority (blue) or after the collision of two fully sorted populations (magenta). Collision assay realized through model wounds. *n* = 8 videos from *N* = 3 independent experiments (Minority); *n* = 6, *N* = 3 (Majority); *n* = 4, *N* = 2 (Collision). **d**, Top: mixed-culture E-cad KO cells (green) and E-cad/cadherin 6 dKO cells (grey). Bottom: area of E-cad KO cells competing against WT (blue) or dKO (green) cells. *n* = 6 videos from *N* = 3 independent experiments (versus WT); *n* = 8, *N* = 2 (versus dKO). **e**, Left: stress map within metaplastic breast tumour tissue, corresponding to **b**. The colour map shows the compressive (blue) and tensile (yellow) stresses. Right: average isotropic stress, *n* = 5 videos from *N* = 2 independent experiments. **f**, Left: stress map (15 kPa, PDMS), corresponding to **c**. Right: average isotropic stress, *n* = 14 videos from *N* = 4 independent experiments. **g**, Left: stress map on soft substrates (370 Pa). Right: average isotropic stress, *n* = 7 videos from *N* = 2 independent experiments. **h**, Area occupied by E-cad KO cells. Green, uncoated glass; magenta, collagen-coated glass; orange, 15 kPa, PDMS, fibronectin (FN) coated; red, 15 kPa, PDMS, collagen coated; blue, 370 Pa polyacrylamide coated with FN. E-cad KO cells are in minority and under tension on stiff and under compression on soft substrates. *n* = 8 videos from *N* = 3 independent experiments (glass); *n* = 7, *N* = 1 (glass, Coll.); *n* = 10, *N* = 3 (15 kPa, Coll.); *n* = 10, *N* = 3 (15 kPa, FN); and *n* = 10, *N* = 3 (370 Pa). **i**, Left: stress map (15 kPa, substrate) corresponding to **d**. Right: average isotropic stress, *n* = 13 videos from *N* = 2 independent experiments. All data points represent the mean value of all the isotropic stresses within one field of view of one frame. Normalization in **c**, **d** and **h** is to the initial area. *P* values are obtained from an unpaired *t*-test. All the magenta lines show the initial cell clusters. Data are presented as mean ± s.d. Scale bars, 200 µm (**a**, **b** and **e**); 100 µm (**c**, **d**, **f**, **g**, **i**). Source data

We set out to investigate if heterogeneities in intercellular adhesion strength and consequently force transmission capabilities could lead to competitive interactions. A pathological example of such molecular heterogeneities can be found in metaplastic breast cancers, a highly aggressive triple-negative breast cancer subtype presenting a therapeutic challenge^31^. The intratumoural heterogeneity in force transmission capability is recapitulated by the presence of at least two sub-populations of cancer cells—epithelial and mesenchymal^31^—with potentially varying E-cad expression levels in the epithelial sub-population^32^. To address how tumour cell subclones sorted and if they competed within a tumour, we cultivated patient-derived xenografts from metaplastic breast cancers and monitored their development. To focus on the role of force transmission capabilities in cell competition, we chose xenografts with a binary state in E-cad expression, that is, in which E-cad is strongly expressed in the epithelial sub-population but absent in the mesenchymal sub-population. The two sub-populations sorted, resulting in clusters of E-cad-positive epithelial cells (E-cad^+^) surrounded by E-cad-negative (E-cad^−^) mesenchymal cells (Fig. 1a). We further observed competition between the cell types: over time, the E-cad^+^ clusters expanded at the cost of E-cad^−^ cells, removing them from the substrate (Fig. 1b and Supplementary Video 1). This increased removal of E-cad^−^ cells was only observed when both sub-populations directly interacted (Supplementary Fig. 1a). We confirmed our observations using cells from a second breast cancer patient (Supplementary Fig. 1b,c and Supplementary Video 2). These observations indeed suggest that heterogeneities in intercellular adhesion strength can lead to cancer cell competition, in which cells with increased adhesion strength win.

**Extended Data Fig. 1:**
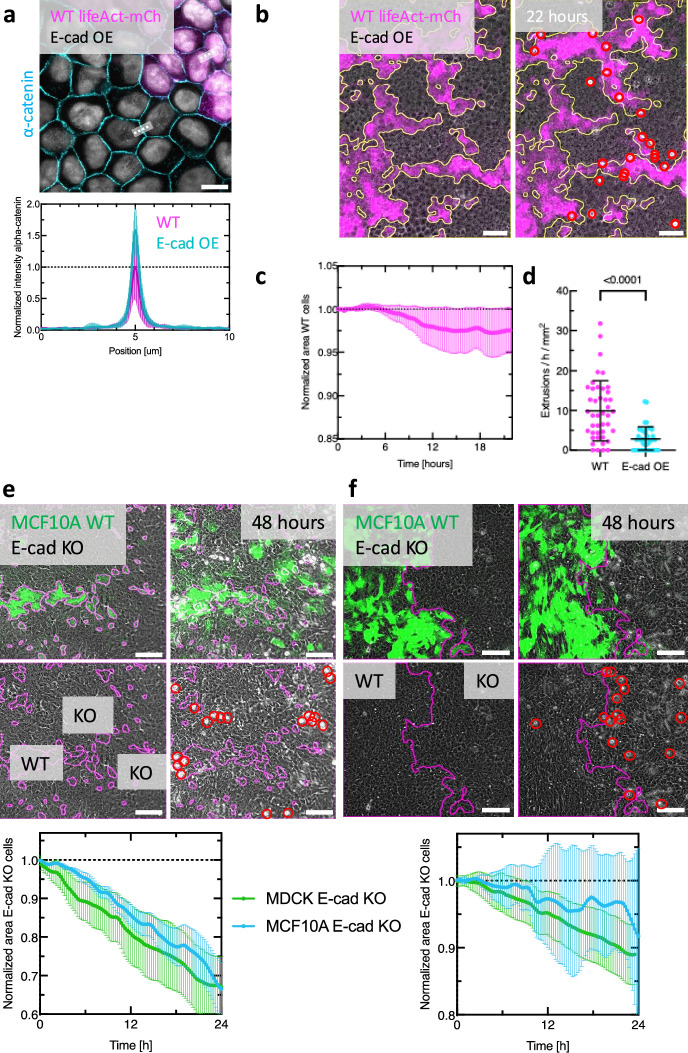
Modulation of intercellular adhesion in MDCK and MCF10A cells. (**a**) Increased alpha-catenin intensity at junctions between E-cad-overexpressing (E-cad OE) cells (described in Adams et al., JCB, 1998) compared to WT cells. Upper: representative confocal image. Lower: Junctional alpha-catenin intensity normalized to WT cells. (**b**) Representative images of mixed culture of MDCK WT lifeAct-mCherry and E-cad OE cells. Yellow outlines show initial clusters, red circles mark extrusions. (**c**) Area development of MDCK WT lifeAct-mCherry cells in competition with E-cad OE cells. n *=* 10 movies from N *=* 2 independent experiments (**d**) Extrusion rates of WT lifeAct-mCherry and E-cad OE cells. Each datapoint shows one time interval from n *=* 4 movies and N *=* 2 independent experiments. P-value from unpaired, two-sided t-test. (**e**) Mixed culture and (**f**) collision of MCF10-A EGFP (green) and MCF10-A E-cad KO (not fluorescent) cells. Magenta outlines show initial clusters. Red circles mark extrusions. Below: Area development of MCF10A E-cad KO cells compared to MDCK E-cad KO cells (shown in Fig. 1) normalized to initial value. MCF10A data: n *=* 5 movies from N *=* 2 independent experiments for coculture and collision. Data are presented as mean values +/− SD. Scale bars 100 µm (C,D); 50 µm (b); 10 µm (a). Source data

To investigate the role of intercellular adhesion in cell competition more systematically, we turned to the competition between two other cell types: we lowered the adhesion strength of MDCK epithelial cells by knocking out E-cad (E-cad KO). In pure cultures, E-cad KO cells showed no signs of reduced cell viability; due to the presence of cadherin 6, they still form mechanically active junctions, although of lower strength^33^. Mixing E-cad KO and wild-type (WT) cells, we observed that the populations sorted^33,34^ and that the E-cad KO cells were outcompeted by the WT cells (Fig. 1c). Because both cell types showed a similar cell density (Supplementary Fig. 2a), we used the change in population area to estimate the cell losses. To quantify the population areas, E-cad KO cells were expressing LifeAct-GFP. These cells lost against normal WT cells as well as WT cells expressing LifeAct-mCherry (Supplementary Fig. 2b), excluding an impact of LifeAct expression on the competition. Importantly, E-cad KO cell loss was independent of the cell ratios, as they also lost when in majority (Supplementary Fig. 2c). To better control the boundary between the two cell types, we developed a collision assay of two migrating cell populations^18,35^, which led to the same competition outcome (Supplementary Fig. 2c and Supplementary Video 3). To assess if and how E-cad KO cells compete against cells with even further reduced cell–cell adhesion, we mixed them with MDCK E-cad/cadherin 6 double knockout cells (dKO), which cannot form any adherens junctions^36^. The previously losing E-cad KO cells won and outcompeted the dKO cells (Fig. 1d and Supplementary Video 4). To modulate the force transmission strength through the expression levels of E-cad, we then mixed WT and E-cad overexpressing^37^ (E-cad OE) cells. WT cells were eliminated by these cells with even further increased cell–cell adhesion (Extended Data Fig. 1a–d). To generalize our findings, we performed similar experiments with another epithelial cell line, which originates from breast tissue, MCF10A cells, mixing WT and E-cad KO cells^38^. The E-cad KO cells were also eliminated by the WT cells, in both mixed cultures (Extended Data Fig. 1e) and collision assays (Extended Data Fig. 1d). Taken together, experiments across diverse cell types show, without exception that cells with relatively stronger adherens junctions always win in cell competition, including patient-derived tumours and various epithelial cell lines.

## Winning cells can be under tensile or compressive stresses

**Extended Data Fig. 2:**
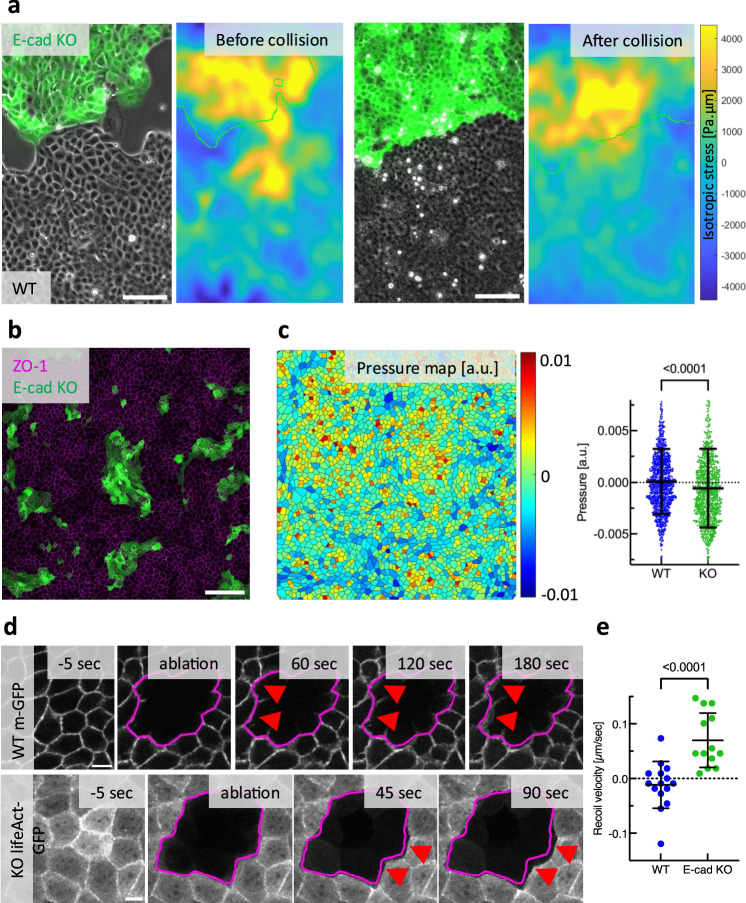
Different validations of stress measurements. (**a**) Isotropic stresses before and after collision of E-cad KO (green) and WT tissues. Before collision, tension is highest at the migration front of both cell types. After collision, the WT cells are under compression. (**b**) Example confocal image of mixed culture (LifeAct-E-cad KO in green) stained for cell boundaries (ZO-1, magenta). (**c**) Example image and average relative pressure inferred based on cell shape, as described in Kong et al., Sci. Rep. 2019. Each datapoint represents a single cell. n *=* 1000 cells representing N *=* 2 independent experiments. (**d**) Representative images of WT cells expressing CAAX-GFP mixed with E-cad KO cells expressing lifeAct-GFP before and after laser ablation. (**e**) Comparison of the recoil velocity measured after the ablation. Each datapoint represents one ablation representing n *=* 15 (WT) and n *=* 13 (E-cad KO) movies from N *=* 2 independent experiments. P-values from unpaired, two-sided t-test. Scale bars 100 µm (a,b); 10 µm (d). Source data

**Extended Data Fig. 3:**
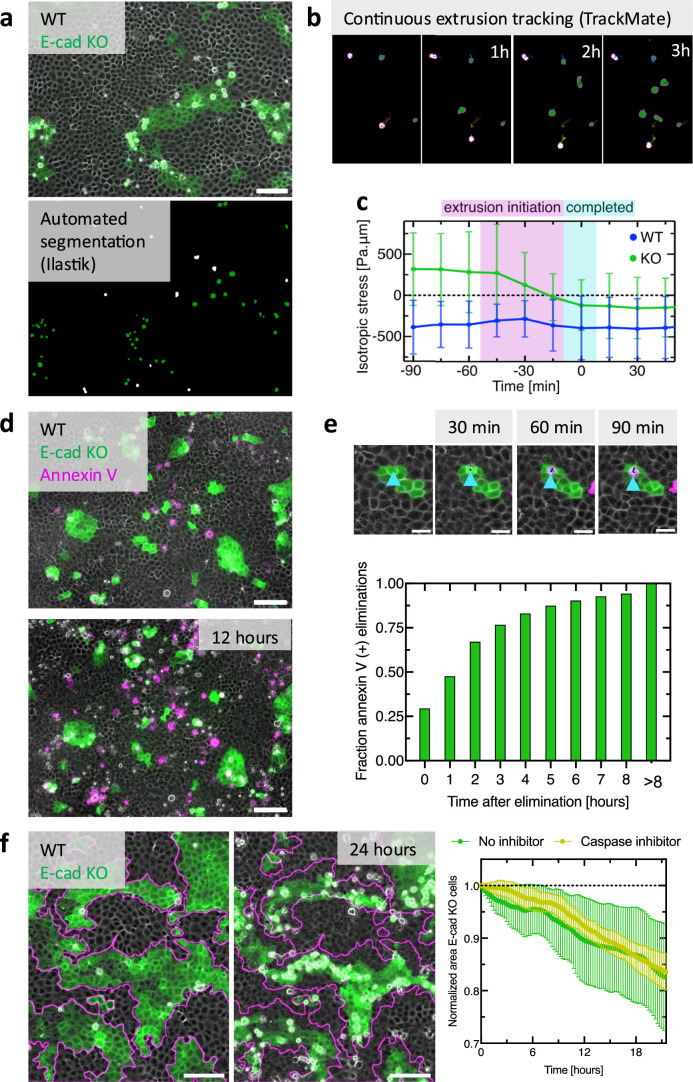
Cell elimination is independent of compressive stress or cell death. To quantify E-cad KO and WT cell extrusions and determine their position, we developed an automated workflow: (***a***) Exemplary raw data (merged brightfield and fluorescent signal) and segmentation of extrusions (white = WT, green = E-cad KO). The supervised learning algorithm ilastik was trained to detect and classify extrusions based on both signals. Details about the training and the accuracy can be found in the Methods section. (***b***) To determine accurate extrusion positions and avoid counting extrusions multiple times, the extrusions were tracked through time using TrackMate. (***c***) Average isotropic stress before E-cad KO and WT extrusions. T = 0 indicates detection, that is completion of the extrusion process. Stresses were measured within a square of size 60 µm around one extrusion event for different time points. n = 726 (E-cad KO) and n = 334 (WT) extrusions in n = 10 movies from N = 2 independent experiments. (***d***) Cell death is detected by the annexin V singal (magenta) in mixed culture. (***e***) Quantification of cell fate. For each extrusion, we calculated the time from cell elimination until detection of the annexin V signal. The plot shows the fraction annexin V-positive cells after the time of extrusion. n = 11327 extrusions from n = 17 movies and N = 4 independent experiments. (***f***) Representative images of mixed culture treated with a pan-caspase inhibitor (Z-VAD-FMK, 20 µM). Magenta line shows initial cluster boundaries. Right: Area quantification compared of the standard experimental condition. N = 1 caspase inhibitor experiment and n = 5 positions. Unless otherwise stated, data are presented as mean values +/− SD. Scale bars 100 µm (*a*, *d*, *f*); 25 µm (*e*). Source data

Cell elimination can be governed in the epithelia by compressive stresses^39–41^. As force transmission within tissues is mainly regulated through adherens junctions^25,27,42^, we first reasoned that stronger intercellular adhesion could allow winning cells to collectively exert compressive stresses on the losing cells, in line with current consensus described in the literature^7–9,11–13,17^. Unlike previous studies, our experimental setup provides additional information that includes direct access to intercellular stresses using Bayesian inversion stress microscopy (BISM)^41,43,44^. In the patient-derived tumour cultures, we observed that the winning E-cad^+^ cells were under high levels of tension and the losing E-cad^−^ cells were under compression (Fig. 1e), in agreement with their strong differences in stiffness (Supplementary Fig. 3a,b) and exerted traction forces (Supplementary Fig. 3c). However, to our surprise, in mixtures of MDCK WT and E-cad KO cells, winning WT cells were under compression and losing E-cad KO cells were under tension (Fig. 1f). This non-intuitive, unanticipated observation is contrary to established models^7–9,11–13,17^. We confirmed this result with the collision assay in which we temporally controlled the establishment of contact between the two cell types. The mechanical state of WT cells switched from tensile during migration to compressive after collision with E-cad KO cells (Extended Data Fig. 2a). Similar results were obtained using another force interference method independent of traction forces and based on cell shape obtained from labelling tight junctions^45^ (Extended Data Fig. 2b,c). They were further confirmed by laser ablation experiments (Extended Data Fig. 2d), where WT cells showed a negative recoil (Supplementary Video 5; compression) and E-cad KO cells showed a positive recoil (Supplementary Video 6 (tension) and Extended Data Fig. 2e). Although E-cad KO cells were on average under tension, local regions remained under compression (Fig. 1f). Thus, we wondered whether E-cad KO cells were preferentially eliminated at these local compressive regions. Assessing the isotropic stresses locally (Extended Data Fig. 3a,b) before cell elimination revealed that E-cad KO cells were under tension before and during the elimination process (Extended Data Fig. 3c). This confirms that the competition outcome is independent of local compressive regions. To further compare this mechanism with previously established cell competition scenarios that include loser cell death^7,9,10^, we investigate the fate of the eliminated cells by labelling dying cells with annexin V. We observed that 70% of E-cad KO cells were eliminated alive and only later died due to their extraction from the tissue and, thus, the absence of adhesion^46^ (Extended Data Fig. 3d,e). Furthermore, we inhibited apoptosis using a pan-caspase inhibitor, which did not change the competition outcome (Extended Data Fig. 3f). Together, these data show that the cell elimination mechanism is independent of loser cell death. Moreover, since cell competition based on biochemical signalling usually leads to cell death^1,2^, live-cell extrusion strongly supports a cell elimination mechanism based on mechanical forces.

To investigate other competition scenarios, we changed the mechanical environment of all cells using softer substrates (370 Pa) to lower the cell–substrate adhesion^44^ (Supplementary Fig. 4a) and exerted tractions (Supplementary Fig. 4b). Under such conditions, E-cad KO cells were now under compression and the WT cells under tension (Fig. 1g), but the competition outcome remained the same, that is, WT cells won independent of substrate composition or stiffness (Fig. 1h). We further measured stresses in the competition between E-cad KO and dKO cells, and observed the same pattern of tension–compression with the winners (E-cad KO cells) under tension and the losers (dKO cells) under compression (Fig. 1i). Overall, we show that compression-induced cell loss can indeed explain the outcome of different competition scenarios. However, the direct measurement of intercellular stresses challenges this established consensus that winners always squeeze out losers. Demonstrating that cells can be under compression and still win suggests that other, still-unknown mechanisms must be governing the cell competition outcome.

## No established mechanism can explain E-cad KO cell elimination

**Fig. 2:**
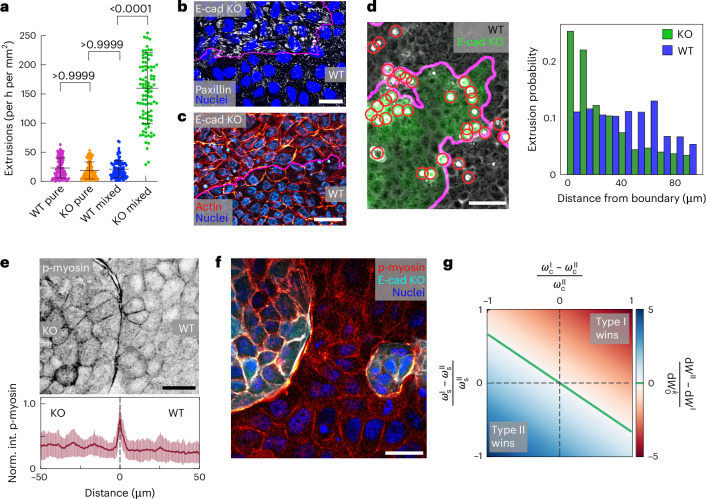
E-cad KO cells are eliminated at a mechanically active interface. **a**, Extrusion rates calculated by counting all extrusions within 1-h intervals normalized to the area occupied by each cell type during the time interval. E-cad KO cells are in minority in the mixed culture. *P* values are obtained from a Kruskal–Wallis test corrected for multiple comparisons (Dunn’s test). Extrusions are quantified for *n* = 100 intervals representing eight videos from *N* = 2 (pure cultures) or *N* = 3 (mixed cultures) independent experiments. **b**,**c**, Relative mechanical properties of E-cad KO cells. Example images of focal adhesions (**b**, paxillin, white) and the actin cytoskeleton (**c**, red, maximum projection). **d**, Left: representative phase-contrast and fluorescence image of MDCK WT and E-cad KO cells (green). The red circles indicate extrusions. Right: probability distribution of cell extrusion regarding the distance from the interface for WT and E-cad KO cells. Cells have a typical diameter of 10–15 µm. *n* = 14,729 KO extrusion and *n* = 11,031 WT extrusion from *N* = 4 independent experiments. **e**, Top: inverted greyscale image of phospho-myosin (p-myosin) at the tissue interface. Bottom: normalized intensity of phospho-myosin (norm. int. p-myosin) obtained from line plots crossing the tissue interface. The lines were centred at the interface (dashed line, distance = 0), indicated by the fluorescence signal from E-cad KO LifeAct-GFP (not shown), averaged from *n* = 24 measurements from *N* = 2 independent experiments. **f**, Example fluorescence image of pluricellular actomyosin cables forming in WT cells observed in co-culture. Cables are enriched in phospho-myosin (red, intensity-coded) and form along islands of E-cad KO cells (cyan). **g**, Phase diagram showing the work required to eliminate cells. The *x* axis shows the difference in cell–cell adhesion. The *y* axis shows the difference in cell–substrate adhesion. The colour code indicates the difference in work, that is, indicates winning and losing. The top left region shows that cells with relatively high cell–cell adhesion can win despite lower relative cell–substrate adhesion. Data are presented as mean values ± s.d. Scale bars, 50 µm (**d**); 25 µm (**c**, **e** and **f**); 10 µm (**b**). Source data

**Extended Data Fig. 4:**
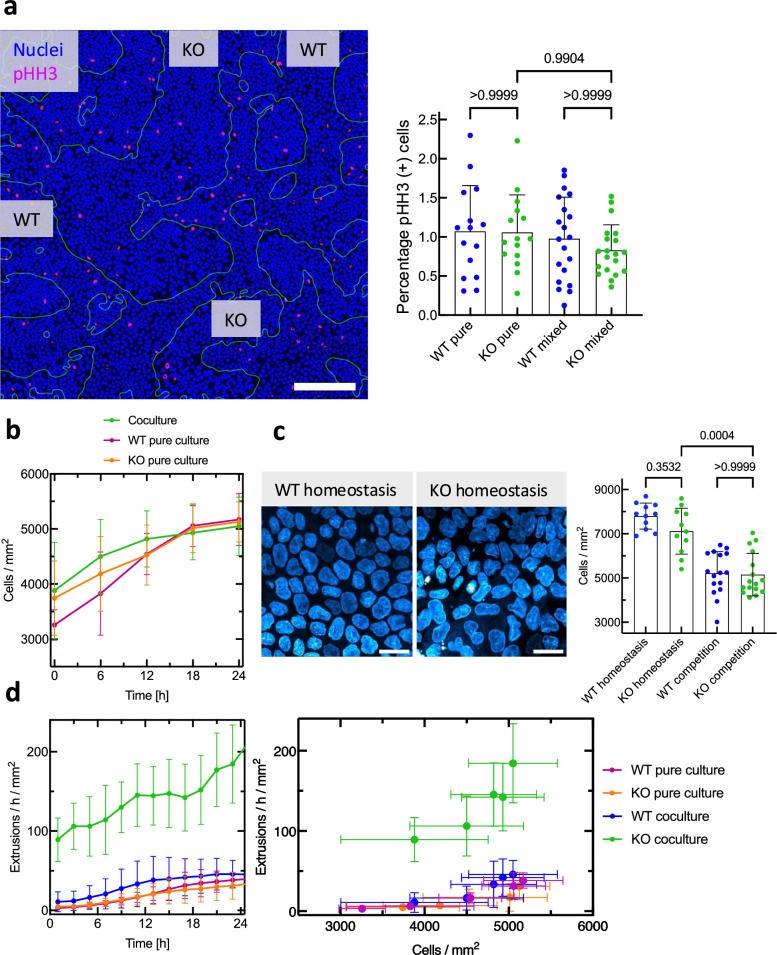
Proliferation, homeostatic densities or crowding cannot explain winning of WT cells. (***a***) Mitotic cells in mixed culture. Segmented nuclei (blue), outlines of E-cad KO clusters (green) and phospho-Histone H3 positive cells (pHH3, magenta) are shown. Right: Average fractions of mitotic cells in pure and mixed cultures. Each datapoint shows the fraction for one FOV. n = 15 FOV from N = 2 independent experiments (pure) and n = 20, N = 2 (mixed). (***b***) Cell densities in pure and mixed cultures over time. n = 7 (pure), n = 10 (mixed) movies from N = 2 (pure), N = 3 (mixed) independent experiments. (***c***) Confocal images of pure WT and E-cad KO cells at homeostasis (4 days confluent culture). Right: Comparison of homeostatic densities to typical densities observed in cell competition (data already shown in Fig. S2 A). Each datapoint shows the fraction for one FOV. n = 11 (homeostasis) and n = 16 (competition) FOV from N = 2 independent experiments. P-value from unpaired t-test. (***d***) Left: Extrusion rates of MDCK cells over time. Rates are calculated for pure and mixed cultures. Right: Extrusion rates over cell densities, corresponding to (***b***). n = 7 movies from N = 2 (pure) and N = 3 (mixed) independent experiments. P-values from Kruskal-Wallis test corrected for multiple comparisons (Dunn’s test). Data are presented as mean values +/− SD. Scale bars (*a*) 200 µm (*b*) 25 µm. Source data

**Extended Data Fig. 5:**
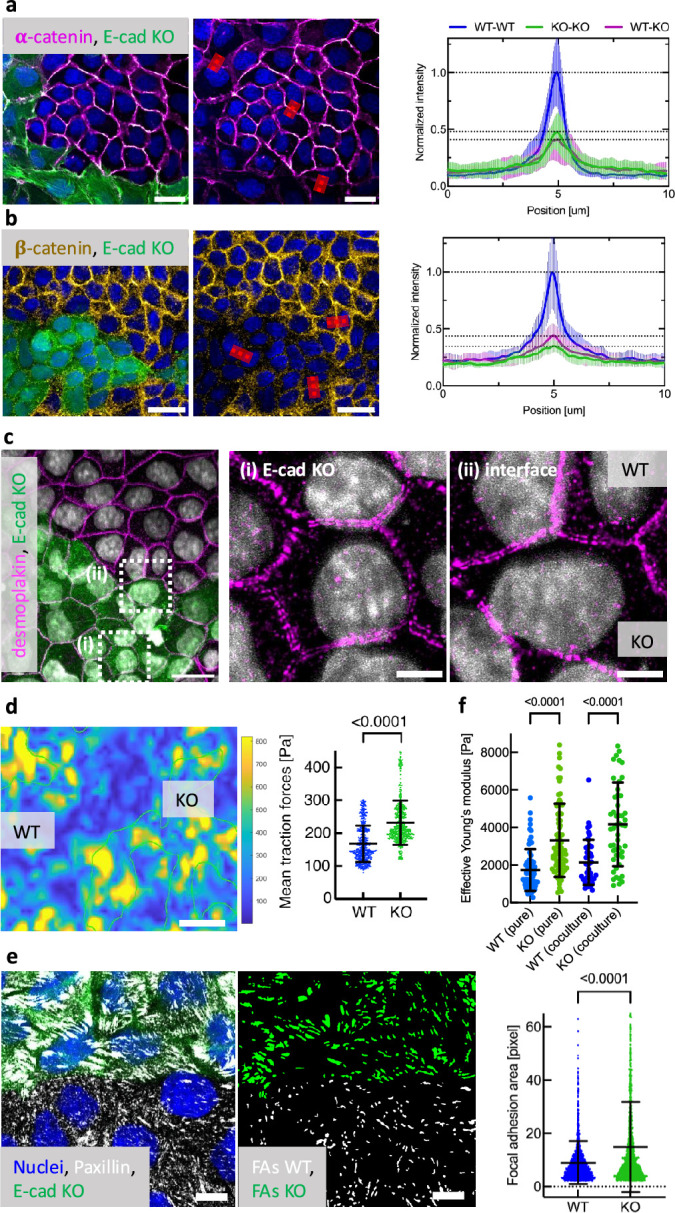
Differences in cell mechanics between MDCK WT and E-cad KO cells in mixed cultures. E-cad KO cells express LifeAct-EGFP and are visualized in green in every image. (***a***) Confocal images showing accumulation of alpha-catenin (magenta) and (***b***) beta-catenin (yellow) at cell-cell junctions. Red lines indicate line plots. Right graphs are normalized to the highest average value. n = 15 (alpha-catenin) and n = 20 (beta-catenin) measurements from N = 1 experiment. (***c***) Airyscan confocal images of desmoplakin showing desmosomes in the mixed cultures. Zoom-ins on (i) the bulk of E-cad KO cells and (ii) on the interface. (***d***) Color-coded traction force map on 15 kPa stiff surface. Green outline shows E-cad KO cluster. Right: Average traction forces. n = 568 positions from N = 4 independent experiments. (***e***) Cell stiffness measured by indentation for pure and mixed cultures. E-cad KO cells in mixed cultures were identified by their green fluorescence. n = 46 (WT), n = 51 (KO) indentations from N = 2 independent experiments. (***f***) Confocal images showing Focal adhesions (Paxillin, white) and corresponding segmentation (middle). Right: Average FA area. n = 1500 FAs representing N = 2 independent experiments. P-values derived from unpaired, two-sided t-test (*d*,*f*) or from Kruskal-Wallis test corrected for multiple comparisons (Dunn’s test) (*e*). Data are presented as mean values +/− SD. Scale bars 50 µm (*d*); 20 µm (*a*, *b*, *c*); 10 µm (*f*); 5 µm (*c*, zoom-ins). Source data

**Extended Data Fig. 6:**
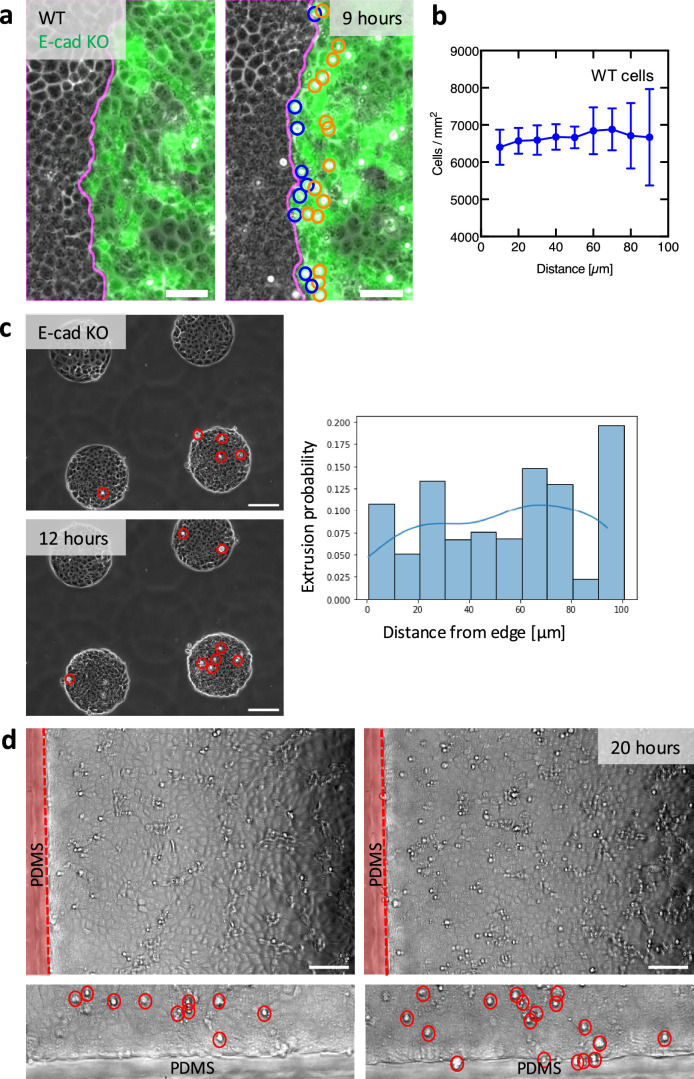
E-cad KO cells do not extrude at a passive interface. (**a**) Example images of E-cad KO cells eliminated at the interface. Cells are eliminated directly in contact with WT cells (blue circles), or close to the interface without directly interacting with the WT cells (orange circles). Magenta line shows the interface. (**b**) Quantification of WT cell density over the distance to the interface based on nuclear segmentation shown earlier (Supplementary Fig. 2a). (**c**) Example images of E-cad KO cells confined on circular micropattern with a 100 µm radius. Red circles indicate extrusions. Below: Extrusion probability of confined E-cad KO cells. n *=* 112 extrusions in N *=* 1 experiment. (**d**) E-cad KO cells confined with a rigid passive fence (indicated by red shade, made of PDMS). Bottom: Zoom-ins on boundary area. Red circles indicate extrusions. No preferred accumulation of extrusions at the interface was observed. Scale bars 100 µm (d), 50 µm (a). Source data

To understand why the E-cad KO cells were losing despite being under tension, we first ruled out previously conjectured mechanisms. For instance, differential cell growth could impact cell competition^10–12^, but both cell types exhibited identical fractions of mitotic cells (Extended Data Fig. 4a) and similar growth rates (Extended Data Fig. 4b) in both pure and mixed cultures. Cells with higher homeostatic density can have a competitive advantage^7,13^, but cell competition emerged at cell densities well below the homeostatic density of both cell types (Extended Data Fig. 4c). Quantifying the rates of cell elimination, both cell types showed similar extrusion rates in pure cultures, and the rates increased with time and cell density (Extended Data Fig. 4d). In mixed cultures, however, the rate of extrusion was strongly increased for E-cad KO cells compared with pure cultures, and independent of cell density, whereas the rate of extrusion for WT cells remained comparable to pure cultures (Fig. 2a and Extended Data Fig. 4d). This demonstrates that the predominant elimination of E-cad KO cells in mixed cultures was not due to intrinsic cell processes but resulted from their collective interactions with WT cells. Previous reports on the role of cell mechanics in cell competition have conjectured that relative increases in cell–substrate adhesion^8,16^ and cell stiffness^12,17^ provide a competitive advantage. Furthermore, E-cad-based adherens junctions have shown to be mechanosensitive, affecting various aspects of cell and tissue mechanics^26,33,47–49^. Thus, we assessed how the decrease in cell–cell adhesion strength (Extended Data Fig. 5a,b) had globally affected the E-cad KO cell mechanics. The cell’s capacity to form tight or desmosome junctions was not changed (Extended Data Figs. 2b and 5c). This underlines that the mechanical link between the cells is only weakened. In mixed cultures, E-cad KO cells exerted significantly larger traction forces on the substratum than their WT counterparts (Extended Data Fig. 5d) and showed a striking increase in the focal adhesion size (Fig. 2b and Extended Data Fig. 5e). Using surface indentation, we measured a significant increase in the E-cad KO cell stiffness compared with WT cells for pure and mixed cultures (Extended Data Fig. 5f), most probably due to their more prominent actin-based contractile phenotype^33^ (Fig. 2c). These observations demonstrate that a cell population’s ability to generate increased forces and exert them on competing cells does not necessarily provide a competitive advantage: loser cells can exhibit stronger cell–substrate adhesions and higher stiffness, which explains the state of tension in eliminated E-cad KO cells, but make their elimination even more puzzling, contradicting the proposed cell–substrate and cell stiffness advantage^8,12,16,17^. Finally, contact-dependent cell–cell signalling could lead to cell elimination independent of mechanical forces^1,2^. However, we observed that E-cad KO cells were eliminated not only at the interface of the two populations but also more than one cell row away from it (Extended Data Fig. 6a and Supplementary Video 7). In conclusion, having tested multiple possibilities, we ruled out the applicability of previously reported mechanisms in explaining the outcome of WT and E-cad KO cell competition, suggesting that a new, hitherto unknown, mechanism must be at play.

## Mechanical interface activity localizes cell eliminations

To further explore the preferred elimination of E-cad KO cells, we investigated the spatial distribution of extrusion events. Previous studies on mechanical cell competition proposed that loser cells get eliminated in the bulk of the cell cluster, where compressive stress is the highest^6^. Moreover, increased contractility at tissue interfaces can impact cell elimination during development^20,22^, but its role remains elusive^21^. We found that the losing E-cad KO cells were preferentially eliminated near the interface, whereas the WT cell extrusions showed a relatively homogeneous distribution (Fig. 2d). The WT cells did not show an increased cell density at the interface (Extended Data Fig. 6b); thus, E-cad KO extrusions were independent of local WT densities. Importantly, neither the free edge of isolated E-cad KO monolayers (Extended Data Fig. 6c) nor an interface of a confined E-cad KO layer with a rigid passive fence (Extended Data Fig. 6d) recapitulated the predominant localization of extrusions at the interface. This suggests that the preferred elimination of E-cad KO cells is triggered by the active interface that emerges between the two tissues with contrasting mechanical properties. Accordingly, the shared interface of E-cad KO and WT cells was strongly enriched in phosphorylated actomyosin in both cell types (Fig. 2e,f and Supplementary Fig. 5a), indicating an increased mechanical activity there. LifeAct and phospho-myosin co-localized (Supplementary Fig. 5a). Using live-cell imaging, we observed a polarization in actin accumulation only at the shared interface at which the cell types first collided (Supplementary Fig. 5b), which underlines the increased interfacial force generation. In this vein, we extended our analysis to the patient-derived tumour cultures. As in MDCK cells, the losing E-cad^−^ cells were extruded at the tissue interface (Supplementary Fig. 5c), at which an increased actomyosin activity was observed (Supplementary Fig. 5d). MDCK WT cells could even form pluricellular actomyosin cables at areas of high negative curvature (Fig. 2f). We hypothesized that the pluricellular formation of actomyosin cables might help WT cells in efficiently removing small E-cad KO clusters through purse-string mechanisms as observed in wound closure^24^. However, E-cad KO cells got eliminated at both regions of positive and negative curvatures (Supplementary Fig. 6). Thus, such cables cannot be a dominant factor here. Independent of curvature, the enrichment in active myosin could generate a mechanical barrier that prevents the mixing of cell types through which they might confine each other^20^. Together, the correlation between cell elimination and high mechanical activity suggests a critical role of this active interface in determining the outcome of cell competition.

We then postulated that cell types endowed with different mechanical properties might react differently to this increased interface activity. To predict how energetically costly it is to eliminate each cell type, we considered a simplified analytical model for energetic requirements of cell elimination: at the interface, two competing cells pull and push on each other, leading to deformations of cells. Thus, the work done on each cell type to deform and eventually eliminate it can be expressed in terms of the energies associated with cell–substrate and cell–cell adhesion strengths, as well as cell stiffness (Methods). The energy required to remove a cell can be simply estimated as the work required to deform the cell from a cylindrical shape to a cone-like shape and then rounding it up to a sphere on cell removal (Supplementary Fig. 7). Comparing the work required to eliminate the competing cells as a function of the difference in their cell–cell adhesion strength demonstrates that the cell type with a higher cell–cell adhesion could require more work to be eliminated, even if the other type has a higher cell–substrate adhesion (Fig. 2g). This simple energetic argument shows that the energy barrier for elimination is higher for cells with strong cell–cell adhesion. As such, this minimal model does not consider where the energy required for elimination comes from and, therefore, does not explain the mechanism driving the elimination. To bridge this gap, we next use a more detailed, cell-based model that resolves individual cells, their interactions and mechanics.

## Stress fluctuations lead to cell elimination in silico

**Fig. 3:**
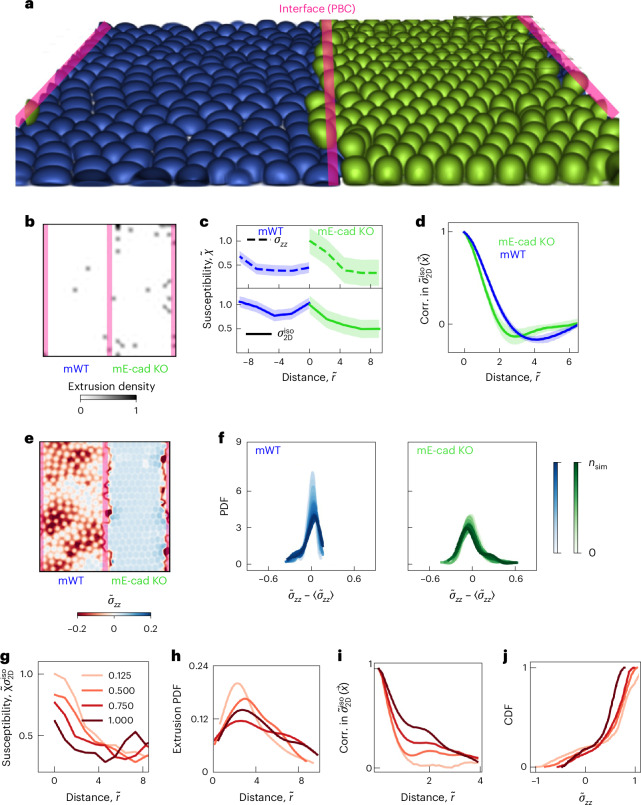
Computational model reveals the role of high fluctuations at the active interface in determining the outcome of cell competition. **a**, Example simulation snapshot with mE-cad KO cells (green) losing to mWT cells (blue) at the interface (red lines), keeping in mind the periodic boundary conditions (PBC). **b**, Extrusion density map representing the spatial distribution of extrusion events, corresponding to the simulation in **a**. **c**, Susceptibility of two-dimensional, that is, in-plane, isotropic stress field and the out-of-plane component of the stress tensor normalized by the maximum value in mE-cad KO cells for each, as a function of distance from the interface. The distance is normalized by the initial cell radius. The data correspond to the simulation in **a**. **d**, Spatial correlation of the in-plane (Corr. in; two-dimensional) isotropic stress for each cell type corresponding to the simulation in **a**. **e**, Out-of-plane stress component field, normalized by the maximum value of in-plane compression. **f**, Probability density function (PDF) for fluctuations in the out-of-plane stress component, normalized by the maximum value of the in-plane compression for each cell type near the interface within the distance of four times the cell radius on each side. The colour shades capture the temporal evolution of the PDFs, where *n*_sim_= 10,000 is the total number of time steps. **g**, Susceptibility of in-plane isotropic stress field for mE-cad KO cells for fixed cell–substrate adhesion and various cell–cell adhesion strengths (${\widetilde{{\rm{\omega }}}}_{{\rm{cc}}}$) normalized by the value for the lowest cell–cell adhesion at the interface. **h**, Extrusion PDFs corresponding to **g**. **i**, Spatial correlations corresponding to coarse-grained in-plane isotropic stress fields averaged (both ensemble and temporal) and centred around an extruding cell in a square domain of eight times cell radius for fixed cell–substrate adhesion and varying cell–cell adhesions corresponding to **g**. **j**, Cumulative distribution function (CDF) corresponding to the average out-of-plane stress fields normalized by the maximum in-plane compression around an extruding cell, showing higher localization for lower cell–cell adhesion: the peak shifts to the left and becomes less tensile as cell–cell adhesion increases. Data are presented as mean values ± s.d. Source data

**Extended Data Fig. 7:**
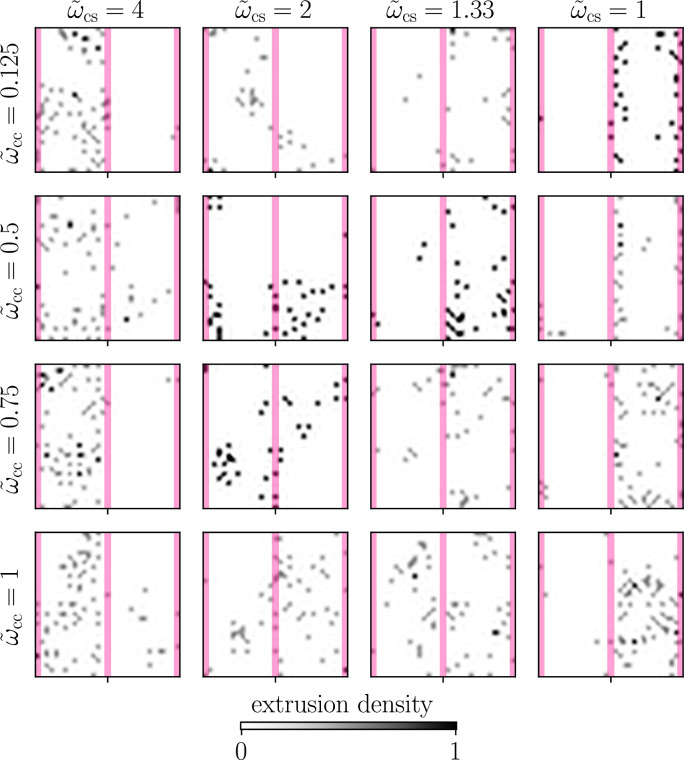
Regulation of type and location of winning cell sub-population by altering their mechanical interactions. Two-dimensional extrusion density maps indicating the location of extrusions for various cell-substrate adhesion strengths ${\widetilde{\omega }}_{{cs}}$ and cell-cell adhesion strengths ${\widetilde{\omega }}_{{cc}}$. The red line indicates the domains associated with mWT cells (left side) and mE-cad KO cells (right side), keeping in mind the periodic boundary conditions. Source data

To understand how the active interface affects mechanical competition and why strong cell–cell adhesion presents a competition advantage, we turned to the physical modelling of three-dimensional (3D) cell monolayers^50,51^. Our model is based on a multiphase field approach that accounts for both passive and active interactions of deformable cells in three dimensions. These interactions include cell–cell and cell–substrate adhesion strengths that are considered explicitly and tuned independently (Supplementary Fig. 8 shows the model schematic). This enables modulating the force transmission capability and its effect on the competition outcome and providing access to the out-of-plane 3D stress components that govern the removal of cells from a monolayer (Methods). Cell extrusion is captured in the model without any explicit threshold, or external artificial means to favour one. Once the out-of-plane forces acting on a cell overpower the forces keeping it in the monolayer and on the substrate, cell extrusion occurs. In this vein, the collective behaviour of cells, for example, cell extrusion and height fluctuations, emerge from solving the dynamics associated with translation and interface relaxation of each cell (Methods and Extended Data Fig. 7). To best represent the experimental conditions, we modelled the collision assays of two model cell types (Fig. 3a and Supplementary Video 8): model WT (mWT) and model E-cad KO (mE-cad KO) defined based on cell–cell adhesion differences (lower for mE-cad KO) and/or cell–substrate adhesion contrast (higher for mE-cad KO). In agreement with the experimental observations, mE-cad KO cells, with a higher cell–substrate adhesion and a lower cell–cell adhesion relative to mWT cells, were eliminated at the interface (Fig. 3b). To understand why E-cad KO cells are eliminated at the interface, we quantified the fluctuations in stress fields via susceptibility^52,53^, which is defined as *χ* = *N* × [〈*σ*^2^〉 – 〈*σ*〉^2^], where 〈〉 indicates expectation and *N* is the number of data points corresponding to *σ* and *σ*^2^ fields. The susceptibility of the isotropic stress field primarily due to in-plane fluctuations ${\chi }_{{\sigma }_{2D}^{{iso}}}$ (Supplementary Fig. 9) and linked to out-of-plane component of stress tensor *σ*_*zz*_ peaked at the interface of mE-cad KO and mWT cells, a consequence of the contrasting physical properties of the cell types considered (Fig. 3c). At the same time, in-plane stress fields exhibited a weaker correlation in mE-cad KO cells relative to mWT cells, suggesting a muted ability to transmit stresses (Fig. 3d). Additionally, *σ*_*zz*_ near the interface exhibited a pronounced localization in mE-cad KO cells relative to their mWT counterparts (Fig. 3e), particularly in the tensile region (Fig. 3f). To further investigate the link between in-plane fluctuations and out-of-plane stress localization, we considered a series of simulations in which the cell–substrate adhesion contrast is kept constant, whereas the contrast in cell–cell adhesion is increased, by reducing the cell–cell adhesion strength of mE-cad KO cells. Interestingly, in-plane susceptibility near the interface decreased (Fig. 3g), whereas the location of extrusion events shifted away from the interface (Fig. 3h) as the contrast in cell–cell adhesion is reduced. These results suggested that higher in-plane fluctuations led to more extrusions of mE-cad KO cells near the interface. To understand why, we focused on stress transmission away from the interface.

We noted a more persistent susceptibility away from the interface, that is, a relatively smaller difference in susceptibility near the interface and further from it, by increasing the cell–cell adhesion (Fig. 3g). More importantly, the characterization of the spatial correlation of averaged, in-plane isotropic stress fields before and at the onset of extrusion (Fig. 3i) showed that these fields became more correlated as the cell–cell adhesion of mE-cad KO cells is increased, signalling a more efficient transmission of mechanical information. Indeed, the inspection of the out-of-plane component of the averaged fields (*σ*_*zz*_) around extrusions show higher localization due to ineffective stress transmission by mE-cad KO cells with low cell–cell adhesion (Fig. 3j), resulting in mE-cad KO cells to extrude near the interface. In summary, the in silico study showed (1) the emergence of an actively fluctuating interface due to differences in cell–cell adhesion strengths and (2) weakening cell–cell adhesion hindering the flow of mechanical information away from this active interface, manifesting in less correlated stress fields. This explained why mE-cad KO cells are eliminated at the interface. Unable to transmit the high in-plane isotropic stress fluctuations away from the interface, mE-cad KO cells seek relief by localizing stresses out of plane and potentially extruding as mWT cells expand into their domain.

## Confirmation of stress fluctuations driving cell elimination

**Fig. 4:**
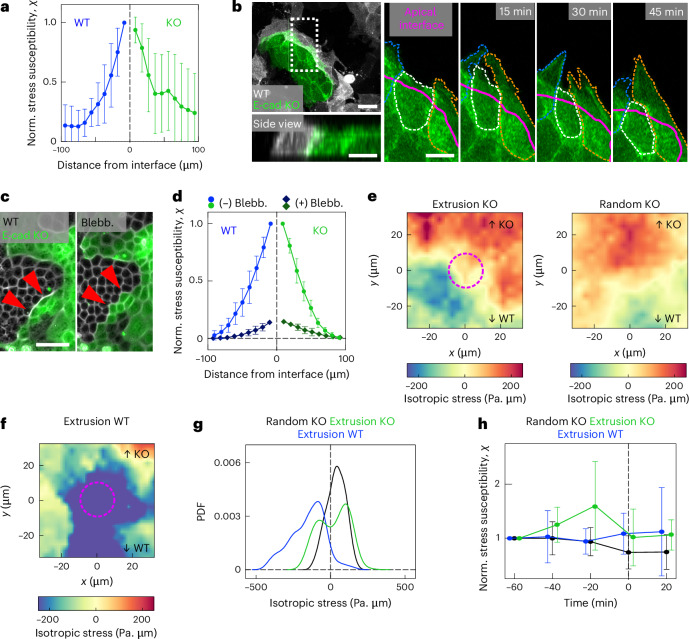
E-cad KO cells are eliminated through increased stress fluctuations. **a**, Isotropic stress susceptibility versus distance from the interface, normalized to the highest value. *n* = 18 videos from *N* = 5 independent experiments including mixed cultures and collisions. **b**, Snapshots of actin dynamics at the interface. Maximum projections and side view of MDCK E-cad KO LifeAct-GFP (green) and MDCK WT LifeAct-Ruby (white). Right: zoomed-in images on the maximum projection of E-cad KO cells protruding below WT cells. The apical interface is indicated by the magenta line drawn based on the WT LifeAct-Ruby signal (not shown). **c**, Phase-contrast and fluorescence images of the boundary (red arrows) before (left) and 2 h after 20 µm blebbistatin (Blebb.) addition (right). **d**, Isotropic stress susceptibility versus distance from the interface for each cell type, before (dots) and after (rectangles) the addition of blebbistatin normalized to the highest value. *n* = 10 videos from *N* = 2 independent experiments. **e**, Left: ensemble average stress heat map before E-cad KO extrusions. Extrusions considered within a 30-µm band from the interface. Stresses were averaged up to 40 min before the automated detection of extrusion, excluding the time point of completed extrusion. Stress fields were oriented (top, KO; bottom, WT) based on the E-cad KO fluorescence signal (Methods). Right: average stress heat map of random positions in E-cad-KO-occupied area at the interface. *n* = 798 extrusions (E-cad KO), *n* = 750 KO random positions from four independent experiments. **f**, Average stress heat map before WT bulk extrusions. *n* = 741 extrusions from *N* = 4 independent experiments. **g**, PDFs of the average isotropic stress distribution before extrusion detection (WT cell bulk elimination, blue; KO cell interface elimination, green; and random KO interface position, black) corresponding to **e** and **f**. **h**, Temporal evolution of the mean isotropic stress susceptibility before (*t* < 0) and briefly after an extrusion event. *t* = 0 indicates the time point of automated extrusion detection. Random position of E-cad KO cells at the interface, black; E-cad KO elimination, green; WT cell elimination, blue. Susceptibility is averaged within a square of size 60 µm around one extrusion event. Normalized to the initial value, *n* = 726 extrusions (E-cad KO), *n* = 1,050 KO random positions and *n* = 334 extrusions (WT) in *n* = 6 videos from *N* = 2 independent experiments. Data are presented as mean values ± s.d. Scale bars, 50 µm (**c**); 10 µm (**b**). Source data

**Extended Data Fig. 8:**
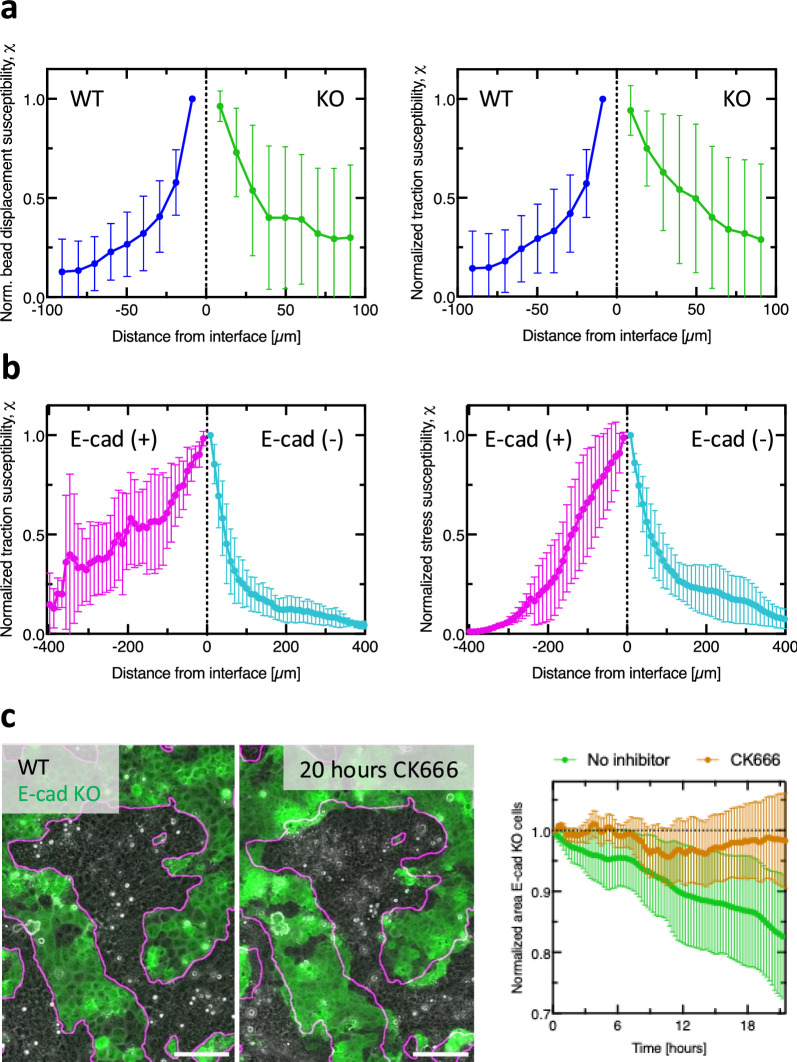
Interface fluctuations occur in MDCK cells and within patient-derived tumor xenograft. (***a***) Susceptibility of bead displacement (left) and of traction forces (right) as a function of distance from the interface in competing MDCK WT and E-cad KO cells. n = 5 movies from N = 2 independent experiments. (***b***) Susceptibility of tractions (left) and stresses (right) as a function of distance from the interface within patient-derived tumor xenograft cultured in 2D. n = 5 movies from N = 2 independent experiments. (***c***) Area development of E-cad KO cells in competition with WT cells in the presence of 100 µM CK666, which inhibits protrusion formation. n = 4 (CK666), n = 10 (no inhibitor) movies from N = 2 independent experiments. Means and standard deviations are shown. Values normalized to maximal value. Scale bars 100 µm. Source data

**Extended Data Fig. 9:**
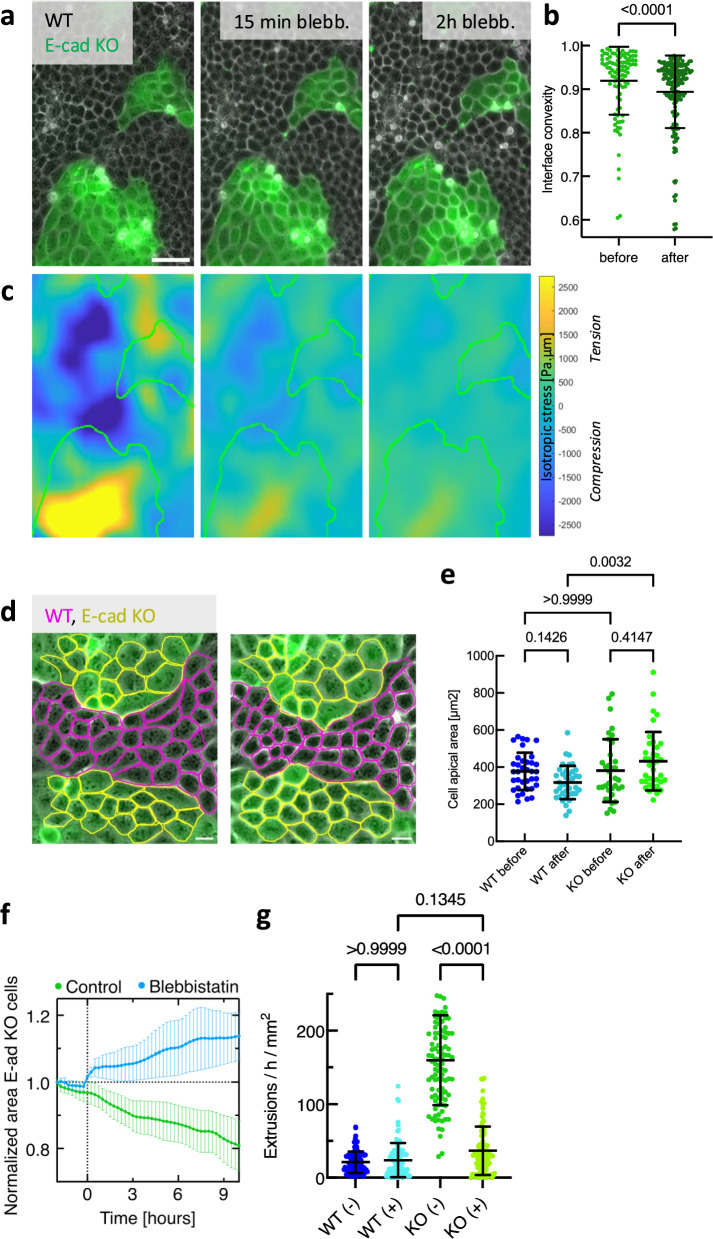
Effect of blebbistatin on cell competition between MDCK WT and E-cad KO cells. (**a**) Additional representative images of the mixed cultures before and after the addition of 20 µM blebbistatin. (**b**) Quantification of the E-cad KO interface convexity before and after blebbistatin addition. P-value from unpaired, two-sided t-test. Data from n *=* 4 movies from N *=* 2 independent experiments. (**c**) Isotropic stress fields before and after blebbistatin addition corresponding to images shown in (a). The red outline shows that WT remain under compression, the magenta outline shows that E-cad KO cells remain under tension. (**d**) Example pictures of apical areas before (left) and 2 h after (right) blebbistatin addition. Apical areas were segmented manually. (**e**) Quantification of cell apical area change after 2 h of blebbistatin addition. n *=* 35 cells from N *=* 1 independent experiment. (**f**) Area development of E-cad KO cells in competition with WT cells. Addition of blebbistatin at t *=* 0 reduces interface fluctuations, which reverses the area development of E-cad KO cells. n *=* 8 movies from N *=* 2 independent experiments. (**g**) Comparison of extrusion rates after 20 µM blebbistatin addition compared to the standard condition. n *=* 100 intervals from N *=* 2 ( *+* blebbistatin) and N *=* 3 (-blebbistatin) independent experiments. P-values from unpaired t-test (b) or Kruskal-Wallis test corrected for multiple comparisons (Dunn’s test) (e, g). Means and standard deviations are shown. Values normalized to maximal value. Scale bar 50 µm. Source data

To verify these predictions, we first experimentally assessed the susceptibility of mechanical stresses and found the same striking increase in stress fluctuations at the interface, which correlates with the localization of E-cad KO extrusions (Fig. 4a). As expected, the increase in fluctuations at the interface was also found in the substrate displacement and in the traction forces (Extended Data Fig. 8a). Additionally, in line with the simulation predictions of enhanced fluctuations at higher cell–cell adhesion difference, we observed even stronger interface fluctuations in the primary tumour sample in which the difference in cell–cell adhesion is higher relative to MDCK cells (Extended Data Fig. 8b). Besides the differences in cell–cell adhesion, we hypothesized that high cellular activity is required for high stress and traction fluctuations. To investigate cellular behaviour at the interface, we assessed the dynamics of the actin cytoskeleton. E-cad KO cells were highly active and extended several micrometre-long protrusions below the surrounding WT cells (Fig. 4b and Supplementary Video 9). This dynamic protrusion activity demonstrated an increased cellular motility of E-cad KO cells at the interface, in line with the increased traction fluctuations. We inhibited protrusion formation using CK-666. E-cad KO cells did not lose any more (Extended Data Fig. 8c), which additionally supports that interface fluctuations are crucial for their elimination. Furthermore, increased mechanical activity could lead to increased stress fluctuations. To reduce the mechanical activity, we treated MDCK cells with blebbistatin. It globally inhibits actomyosin-generated cellular forces, which might have variable effects on the entire cell population. Blebbistatin disrupted the interface between the cells, evident by a reduced interface convexity (Fig. 4c and Extended Data Fig. 9a,b). Importantly, blebbistatin decreased the stress magnitudes (Extended Data Fig. 9c), leading to cell relaxation^54^. Due to their increased contractility, this relaxation is relatively stronger in E-cad KO cells, increasing the area of single cells (Extended Data Fig. 9d), and leading to an E-cad KO domain increase after blebbistatin addition (Extended Data Fig. 9e). The reduction in cellular forces led to a striking drop in stress fluctuations (Fig. 4d), which correlated with a significant reduction in the global extrusion rate of E-cad KO cells, whereas the extrusion rate for WT cells remained the same (Extended Data Fig. 9f). These experiments confirm the emergence of increased stress fluctuations at mechanically active tissue interfaces and indicate that maintaining the active interface is required for WT cells winning.

**Extended Data Fig. 10:**
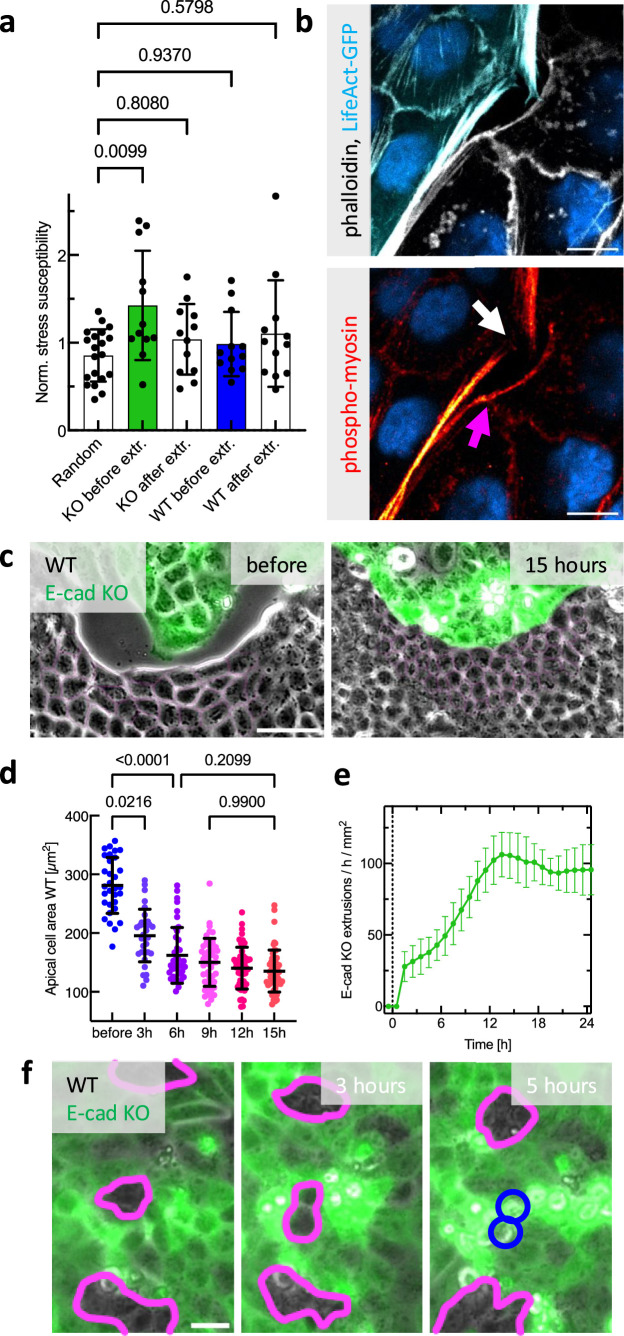
Force transmission and collective deformation provide a competitive advantage. (**a**) Comparison of the normalized stress susceptibility corresponding to Fig. 4h. Random positions of E-cad KO cells at the interface, E-cad KO and WT before (t *=* −40 and t *=* −20 in Fig. 5h) and after the extrusion event (t *=* 0 and t *=* 20) are shown. n *=* 12 (KO, WT) and n *=* 20 (Random) time intervals from N *=* 2 independent experiments. (**b**) Exemplary airyscan image of contractile actomyosin cables at the interface. Actin in white, phospho-myosin in red. Actomyosin fibers are interrupted between E-cad KO cells (white arrow) but can span multiple cells between WT cells (magenta arrow). (**c**) Phase contrast images of WT and E-cad KO cells colliding. Magenta outlines show manual area quantification of WT cells. (**d**) Manual segmentation of the WT apical area following tissue collision. Each datapoint represents one cell. n *=* 31 (before) to n *=* 54 (15 h) cell, as they migrate into the FOV or divide. Data from N *=* 1 preparation. (**e**) Quantification of the extrusion rate over time. T *=* 0 indicates the timepoint of collision. n *=* 4 movies from N *=* 2 independent experiments (**f**) Example of WT cell doublet being eliminated (blue circles) by surrounding E-cad KO cells. P-values from Kruskal-Wallis test corrected for multiple comparisons (Dunn’s test). Means and standard deviations are shown. Values normalized to maximal value. Scale bar 50 µm (c), 20 µm (f), 10 µm (b). Source data

To further explore the relationship between interface stress fluctuations and cell elimination, we assessed the local stress fields before cell extrusions close to the interface by computing the ensemble average stresses up to 40 min before the extrusion event. The stress field around the cell extrusion events in E-cad KO cells exhibited high values of both compressive and tensile stresses (Fig. 4e, left), whereas the one at random positions at the interface were under lower values of tensile stresses (Fig. 4e, right). This indicates that E-cad KO cells experienced increased fluctuations of stresses before their elimination at the interface. By contrast, the stress field around extruding WT cells, which showed no preference for being eliminated at the interface (Fig. 2d), was exclusively compressive (Fig. 4f). These findings are confirmed by the distribution of isotropic stresses, which showed a much wider range and more extreme values of both compressive and tensile stresses for E-cad KO cells destined to extrude compared with E-cad KO cells at random positions (Fig. 4g). We next analysed the temporal evolution of local stress fluctuations up to 60 min before extrusion and compared them with fluctuations at random positions along the interface. Although the fluctuations at random positions and for WT cells remained relatively stable, we observed a strong and significant increase starting 40 min before E-cad KO cell extrusion events (Fig. 4h and Extended Data Fig. 10a). After removal, these fluctuations returned to the initial level (Fig. 4h). Together, these different mechanical signatures of cell elimination point towards different cell elimination mechanisms: WT cells are extruded through high compressive stresses^39–41^. By contrast, we found another cell elimination mechanism as E-cad KO cells are eliminated at the interface through increased stress fluctuations. In the competition between two cell types, this latter mechanism based on stress fluctuations can be dominant and governs the outcome.

## Collective stress transmission prevents cell elimination

**Fig. 5:**
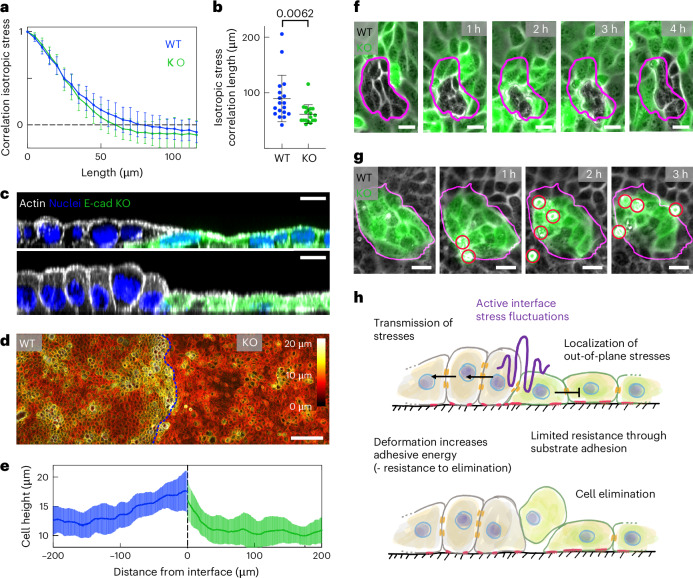
Increased intercellular adhesion endows cells with increased resistance to elimination. **a**, Spatial autocorrelation of the isotropic stress for each cell type. The zero crossing indicates the correlation length. *n* = 18 videos from *N* = 5 independent experiments. **b**, Average correlation length of the isotropic stress for each cell type. *P* values were obtained from an unpaired *t*-test. *n* = 18 videos from *N* = 5 independent experiments. **c**, Deformation of WT cells close to the interface. A confocal image showing the side views of actin (white), LifeAct-GFP in E-cad KO cells (green) and the nuclei. Top: example cell height in mixed culture. Bottom: infrequent increased height for WT cells. **d**, Colour-coded height projection of the actin signal in collision assay. **e**, Quantification of cell height in collision. E-cad KO cells exhibits a faster decrease in cell height with distance from the interface compared with WT. *n* = 15 positions from *N* = 2 independent experiments. **f**, Bright-field and fluorescence (E-cad KO, green) images of WT cells getting compacted without cell elimination. **g**, E-cad KO cells responding to island compaction through cell elimination. **h**, Sketch of the proposed mechanism. Data are presented as mean values ± s.d. Scale bars, 100 µm (**d**); 20 µm (**g**); 10 µm (**c** and **f**). Source data

Cells at the interface were subjected to increased stress fluctuations, but only the ones with lower intercellular adhesion were eliminated. Therefore, we reasoned that high intercellular adhesion must endow the winning ones with mechanisms to resist stress-fluctuation-mediated elimination. The computational model predicted more efficient stress transmission to neighbouring cells, preventing the localization of out-of-plane stresses in winning cells (Fig. 3d–f). Indeed, WT cells showed a significantly increased the stress correlation length compared with E-cad KO cells (Fig. 5a,b). This confirms a more efficient transmission of mechanical stress to neighbouring cells for WT cells. The observation of multicellular actomyosin cables between WT cells (Fig. 2f) but not between E-cad KO cells (Extended Data Fig. 10b) supports these measurements. Furthermore, we reasoned that the proposed mechanism of stress fluctuations at the interface should be reflected in deformations and changes in cell shape. To this end, we assessed the cell height and the cell–cell adhesion area of WT and E-cad KO cells. Although both cell types normally have the same height (Fig. 5c, top), cell shapes fluctuated near the interface; in particular, WT cells could morph into a columnar shape (Fig. 5c, bottom). The differences in cell shapes on the collective level were the most striking in the collision assay, where the WT cell deformation started from the boundary and extended over multiple cells into the bulk. However, the E-cad KO cells did not deform collectively and cells away from the interface remained flat (Fig. 5d,e). The WT cells strongly deformed within the first 12 h following collision (Extended Data Fig. 10c,d), which correlated well with the increasing E-cad KO extrusion rate (Extended Data Fig. 10e). Moreover, when surrounded by E-cad KO cells, islands of WT cells could collectively sustain high deformation and drastic cell area fluctuations over hours without being extruded (Fig. 5f). Such changes in cell shape, thus, increased the intercellular contact zone between WT cells, allowing them to further increase their adhesive energy to better resist stress fluctuations. In particular, some doublets of WT cells were eliminated by E-cad KO cells, suggesting that isolated WT cells cannot propagate stresses and lose their advantage (Extended Data Fig. 10f). The mirror situation revealed that islands of E-cad KO cells did not undergo such strong deformations and released stresses through cell elimination by extrusion (Fig. 5g). Together, these experiments confirmed enhanced stress transmission in winning cell types and further show that keeping strong intercellular adhesion allows the winning type to resist elimination through substantial cell shape deformations.

## Outlook

Here we discover that differences in the force transmission capability directly determine the outcome of mechanical cell competition, in which cells with stronger intercellular adhesion are exclusively winning. Because no previously described cell elimination mechanism could explain our observations, we propose a new one based on combining simulation and experiments. We note that a possible contribution of secreted, extracellular factors to cell elimination^1^ cannot be completely excluded. Comparing stress patterns across multiple competition scenarios demonstrates that cells with increased force generation are under tension, which compresses the other cell population. Because these stress patterns cannot predict winning and losing, we propose that the force transmission—rather than force generation—capability governs the competition outcome. Thus, our proposed mechanism is independent of loser cell compression^7–9,17,20^ and differences in growth rate or homeostatic density^11–13^. Increased fluctuations of isotropic stresses emerge at active interfaces between tissues with different mechanical properties. These high fluctuations in local stress fields near the interface, if not transmitted efficiently by the front-line cells to the rest of the collective, localize and induce out-of-plane stresses, akin to the Poisson effect in elasticity, which can lead to cell elimination. In scenarios where cells with heterogeneities in the force transmission capability compete against each other, intercellular adhesion provides a generic winning strategy because it enables winning cells to withstand higher fluctuations of stresses than losing cells. Thus, unlike other forms of mechanical cell competition such as directed migration towards losing cells^7,8,15^, our findings unveil an alternative mechanism based on active resistance to elimination through a reinforcement of intercellular adhesion. Indeed, cells with higher intercellular adhesion can transmit stresses more efficiently to neighbouring cells, which prevents the localization of elimination-promoting out-of-plane stresses. In addition, increased intercellular adhesion allows collective changes in cell shape into a columnar shape, which increases the mechanical threshold required for elimination by further increasing the adhesive energy. By contrast, cells with relatively lower intercellular adhesion are eliminated through the localization of high-stress fluctuations at the interface and an overall limited resistance to out-of-plane stresses (Fig. 5h). Our conclusions are based on a physical model, only relying on the effect of mechanical imprints. Thus, if similar mechanical imprints are given, this proposed framework could have important implications for different biological processes beyond cell competition. Since it does not rely on loser cell death, it could have a role in organizing tissues during morphogenesis. Differences in force transmission could be involved in maintaining tissue boundaries and, thus, functionality in homeostasis. In the skin, for example, loser cells are expelled apically through an unknown mechanism, and failed competition leads to deteriorated barrier function^55^. The reduction in intercellular adhesion has been associated with metastasis for a long time^56^. Adding to these mechanisms, increased stress fluctuations at the interface of tumoural and normal tissues could also have a role in the invasion mechanism or promote metastasis, if tumoural cells are eliminated alive^6,57^. Moreover, as suggested by our experiments using patient-derived tumour xenografts, this mechanism of cell competition could be acting within tumours with heterogeneities in their cell–cell adhesion strength. Although this study is mainly focused on the binary expression of adhesion molecules, further studies need to address the heterogeneity of cadherin expression levels, which are present in other breast cancers^32^. Mechanical cell competition might change the fate of cells, that is, promote invasion and subsequent metastasis of sub-populations. Thus, it will be exciting to further explore the role of this form of cell competition in tissue sculpting and different pathologies.

## Methods

### Substrate preparation

#### Polyacrylamide gel preparation

Here 370-Pa soft polyacrylamide gels were prepared as described previously^58^. In brief, glass coverslips were cleaned in an ethanol bath, sonicated for 1 min and dried at 80 °C for 15 min afterwards. The coverslips were treated with high-power plasma in a plasma cleaner for 10 min. Then, they were soaked in a silane solution consisting of 2% 3-(trimethoxysilyl)propylmethacrylate (catalogue no. 440159, Sigma-Aldrich) and 1% acetic acid in ethanol for 30 min. The silanized coverslips were rinsed with ethanol, dried at 80 °C for 1 h and stored at room temperature.

Plasma-cleaned glass coverslips or plasma-cleaned glass-bottom imaging dishes (FluoroDish, WPI) were incubated with 5% fibronectin (Sigma) in phosphate-buffered saline (PBS) and dried at 4 °C. A freshly prepared polyacrylamide solution (3% acrylamide (catalogue no. 161-0140, Bio-Rad), 0.06% bis-acrylamide (catalogue no. 161-0142, Bio-Rad), 0.05% ammonium persulphate (catalogue no. 161-0700, Bio-Rad), 0.15% TEMED, in PBS) containing 4% fluorescent beads (FluoSpheres, Invitrogen) was sandwiched between the silanized and fibronectin-coated glass coverslips and polymerized at room temperature for 15 min, resulting in a 100-μm-thick gel. The substrate was kept in PBS at 4 °C.

#### Traction force microscopy

Here 15-kPa soft silicone substrates for traction force microscopy (TFM) were prepared as described previously^3^. In brief, CY52-276A and CY52-276B polydimethylsiloxane (PDMS; Dow Corning Toray) were mixed in a weight ratio of 1:1 (15 kPa) and then poured on glass-bottom imaging dishes (FluoroDish, WPI) to obtain a layer with a thickness of around 100 µm. The gel-covered dishes were spin coated for 60 s at 400 rpm. The substrates were then cured at 80 °C for 2 h. Before seeding of the beads, the surface was silanized using a solution of 5% (3-aminopropyl)triethoxysilane (Sigma-Aldrich) diluted at 10% in absolute ethanol for 10 min and then washed with absolute ethanol three times, before being dried at 80 °C for about 10 min. Here 200 nm red carboxylated fluorescent beads (FluoSpheres, Invitrogen) were diluted at a 2:1,000 ratio in water, and subjected to an ultrasonic bath for 10 min. The bead solution was then filtered using a 0.22-µm filter and incubated on the substrates for 15 min, protected from light. The dishes were finally washed with water three times and dried at 80 °C for 3 min. Before cell seeding, these substrates were coated with 50 µg ml^–1^ fibronectin or collagen (Sigma) for 45 min and washed three times with PBS. Adding Cy3-labelled fibronectin shows a uniform surface coating (Supplementary Fig. 10).

#### Micropatterning

PDMS stamps for micropatterning were prepared as described previously^4^. Moulds of the desired pattern were obtained using standard lithography methods. PDMS (SYLGARD 184, Dow Corning) was prepared by mixing the base with a curing agent at a ratio of 1:10, poured over the mould, degassed and then cured at 80 °C for 2 h. Stamps were peeled of the mould and stored, protected from light and humidity. On utilization, the stamp surface was activated using plasma cleaning to make it hydrophilic, and a mixture of Cy3-conjugated fibronectin and regular fibronectin (50 µg ml^–1^, Sigma) was then incubated covering the whole surface for 45 min, after which the surface was cleaned using a gentle air flow. The stamps were gently pressed against the bottom of a PDMS-covered culture dish for about 1 min for the pattern to imprint and with the stamps carefully lifted; the Petri dishes were rinsed using PBS. The integrity of the patterns was verified using epifluorescence microscopy (Nikon). Patterns were then incubated with a solution of 2% Pluronic F-127 (Sigma) for 1 h to prevent cells from adhering on the unstamped areas. The Petri dishes were rinsed using PBS before seeding the cells.

### Cell culture

The following cell lines were used:

- MDCK-II (ATCC CCL-34)
- MDCK-II LifeAct-Ruby^59^
- MDCK-II E-cad KO (clone B6P6 (ref. ^33^))
- MDCK-II E-cad KO LifeAct-EGFP (clone B6P6 (ref. ^33^))
- MDCK-II E-cad/cadherin 6 dKO (clone D5 (ref. ^36^))
- MDCK-II E-cad overexpression (MDCK E-cad GFP^37^).
- MCF10A EGFP^60^
- MCF10A E-cad KO^38^

MDCK-II cells were cultured in Dulbecco’s modified Eagle’s medium (DMEM; GlutaMAX, high glucose and pyruvate, Life Technologies) supplemented with 10% fetal bovine serum (FBS, Life Technologies) and 1% penicillin–streptomycin (Life Technologies) at 37 °C with 5% CO_2_. MCF10A cells were maintained in DMEM-F-12 (no. 11039-021, Gibco) containing 10% penicillin–glutamine, 10 μg ml^–1^ human insulin (no. I9278, Sigma-Aldrich), 100 ng ml^–1^ cholera toxin (no. C8052, Sigma-Aldrich), 0.5 mg ml^–1^ hydrocortisone (Sigma-Aldrich), 5% horse serum and 20 ng ml^–1^ EGF (PeproTeck) at 5% CO_2_ in an incubator at 37 °C. Cells were passaged every 2–3 days using 0.05% trypsin (catalogue no. 9002077, Merck). Before processing, the culture medium was aspirated and cells were rinsed with PBS to remove dead cells and debris.

### Sample preparation

#### Co-culture experiments

Competing cells were mixed in a suspension using different ratios, seeded on a glass-bottom imaging dish (FluoroDish) or a glass-bottom imaging dish coated with 30 µg ml^–1^ collagen G (type 1, from calf skin, catalogue no. L7213, Sigma-Aldrich) or a PDMS or polyacrylamide substrate for TFM, and cultivated under standard conditions. After the desired cell density was reached, the culture medium was aspirated and cells were rinsed with warm PBS.

#### Collision experiments

Here 5 × 10^4^ cells were seeded in 80 μl culture medium into the wells of a two-well culture inlet (catalogue no. 81176, ibidi) on a glass-bottom culture dish or a PDMS substrate for TFM. The cells were grown overnight and the inlet was removed to allow the different populations to migrate towards each other.

#### Ex vivo culture of tumour-patient-derived xenografts

Breast-cancer-patient-derived xenografts were obtained from triple-negative metaplastic breast tumours (HBCx-60 and HBCx-90) and generated as previously described^61^. After surgical excision of the tumour xenograft, tumouroids were isolated as previously described^61^. Briefly, tissues were cut into small pieces and digested in RPMI 1640 medium supplemented with 4 mg ml^–1^ collagenase (Sigma-Aldrich) in 10 mM HEPES, 5% FBS, penicillin–streptomycin (1×) and glutamine (1×) for 1 h at 37 °C on a rotating wheel at 150 rpm as previously described. Tumouroids were pelleted at 400*g* for 10 min. Then, the tumouroids were incubated for 3–5 min at room temperature in DMEM/F-12/DNase (2 U μl^–1^). Tumouroids were pelleted at 400*g* for 10 min. To remove the fibroblasts, tumouroids were washed with DMEM/F-12 medium and centrifuged at 400*g* for 3 s at room temperature until the supernatant was clear. Tumouroids were resuspended in DMEM with 10 mM HEPES, 5% FBS, 5 μg ml^–1^ insulin, 10 ng ml^–1^ cholera toxin, 1 mg ml^–1^ hydrocortisone, penicillin–streptomycin (1×) and glutamine (1×). Around 800 tumouroids were plated on a FluoroDish coated with fibronectin (50 µg ml^–1^) 37 °C. Metaplastic breast cancers contain epithelial (E-cad^+^, vimentin^–^) and mesenchymal (E-cad^–^, vimentin^+^) cancer cells.

### Stiffness measurements

Nanoindentation was used to measure the stiffness of the cell monolayers. The indenter (Chiaro nanoindenter, Optics11 Life) was connected to an epifluorescence microscope equipped with a ×20 objective to visualize the indentation position. A soft probe with a small tip (*k* = 0.015 N m^–1^; tip radius, 3 μm) was calibrated on glass before the measurement according to the manufacturer’s protocol.

Cell monolayers (pure MDCK WT, pure MDCK E-cad KO, co-culture of WT and E-cad KO LifeAct-EGFP) were grown on glass and measured after confluency. Tumours were grown on fibronectin-coated glass or a thick layer of collagen. Before every measurement, the distance to the surface of the sample was automatically determined, and the probe was placed 5 μm above the surface. To measure the stiffness, the probe was pushed 1.5 μm into the sample for 4 s and retracted afterwards. The matrix scan function was used with a typical step size of 25 μm and time-lapse videos were acquired to trace back the indentation positions. For co-culture measurements, E-cad KO LifeAct-GFP clusters were identified using the epifluorescence signal and the indentation positions were adjusted accordingly.

To determine the elastic modulus, the loading curve was analysed using the built-in software (DataViewer V2, Optics11 Life). The analysis is based on the Hertz model (Hertzian contact), which assumes a linear elastic response of the sample. The single-fit method was used with a maximum load (*P*_max_) of 90% and a Poisson’s ratio of 0.5. Loading curves, which started in contact due to a failed determination of the surface, were automatically excluded from the analysis.

### Time-lapse microscopy

Confluent co-culture monolayers or colliding cell populations were rinsed with warm PBS and a fresh cultivation medium was added. For experiments in which a pan-caspase inhibitor or an inhibitor of cell protrusions was used, 20 µm Z-VAD-FMK (catalogue no. tlrl-vad, InvivoGen) or 100 µM CK-666 were added before starting the experiment. For experiments in which actomyosin contractility was inhibited, 20 μM blebbistatin (catalogue no. 203390, Sigma-Aldrich) was added to the cultivation medium during the experiment.

The dish was transferred to a live-cell epifluorescence microscope (BioStation IM-Q, Nikon, equipped with a ×10 or ×20 phase-contrast air objective and an incubation chamber) and incubated at 37 °C and 5% CO_2_. The monolayer was imaged using phase contrast, and the E-cad KO LifeAct-EGFP population and fluorescent beads were imaged using epifluorescence. Time-lapse videos were taken at multiple positions every 15 min. In the case of TFM, cells were also removed at the end of the experiment by adding 200 µl of 10% sodium dodecyl sulfate in the medium to obtain the relaxed state of beads on the substrate.

### Laser ablation experiments

MDCK WT and E-cad KO cells were seeded on a glass-bottom imaging dish at a ratio of 50:50 and grown until reaching confluency such that large islands of each cell type could be observed. Before wound induction, dishes were rinsed using warm PBS and provided with a fresh culture medium. Laser ablation was done using a spinning-disc CSU-X1 with a fluorescence recovery after photobleaching module (Yokogawa) and a ×40/1.2 water-immersion objective. Briefly, holes with a size of 3–4 cells were inducted within the area of the same cell type in the mixture, focusing an ultraviolet laser (355 nm, pulse duration of 3–5 ns and laser power of 450 nW) for 1 s. Each sample was imaged during 15 s before ablation and until 3 min after ablation, using 5-s intervals. The recoil velocity was measured by manually segmenting the edge holes over time and plotting the change in displacement of the edges of the ablated region.

### Indirect immunostaining

Co-culture or collision experiments that reached the desired cell density were rinsed with warm PBS. Fixation was carried out in 4% paraformaldehyde for 10 min at room temperature. Cells were permeabilized using 0.1% Triton X-100 in PBS for 5 min followed by 5-min washing in PBS three times. Samples were blocked with 1% bovine serum albumin and 10% FBS in PBS for 1 h at room temperature. It is noteworthy that the patient-derived xenograft tumouroid cells were permeabilized using 0.1% Triton X-100 in PBS for 10 min. All the following primary antibodies were diluted at 1:100 in a blocking solution and incubated for 2 h at room temperature or overnight at 4 °C.

- nti-E-cad mouse antibody (catalogue no. 610181, BD Biosciences)
- Anti-E-cad clone ECCD2 for the two-dimensional patient-derived xenograft staining (catalogue no. 1319000, Thermo Fisher)
- Anti-α-catenin rabbit antibody (catalogue no. AB51032, Abcam)
- Anti-β-catenin rabbit antibody (catalogue no. 610156, BD Biosciences)
- Anti-paxillin rabbit antibody (catalogue no. AB32084, Abcam)
- Anti-phospho-myosin light chain 2 rabbit antibody (catalogue no. 3671S, Cell Signaling)
- Anti-ZO1 rabbit antibody (catalogue no. 402300, Life Technologies)
- Anti-vimentin antibody (catalogue no. 8978, Thermo Fisher)
- Anti-phospho-histone H3 mouse antibody (Ser10, catalogue no. 9706, Cell Signaling)
- Anti-desmoplakin mouse antibody (catalogue no. Cl.11-5F, Sigma)

The samples were washed for 5 min in PBS three times and incubated with an anti-rabbit (catalogue no. A31573, Life Technologies) or an anti-mouse (catalogue no. A31571, Life Technologies) antibody conjugated to Alexa Fluor 647 diluted at 1:200 in a locking solution for 2 h at room temperature. Subsequently, samples were washed for 5 min in PBS three times. The actin cytoskeleton was visualized using phalloidin-Alexa Fluor 568 (catalogue no. A12380, Life Technologies) diluted at 1:200 in PBS and the nuclei were visualized using Hoechst 33342 (catalogue no. 62249, Thermo Fisher) diluted at 1:2,000 in PBS for 45 min at room temperature.

### Confocal microscopy and data visualization

Fixed samples were mounted on a laser scanning confocal microscope (ZEISS LSM 980), equipped with a ×63 oil objective and an Airyscan 2 module. MDCK WT cells expressing LifeAct-mCherry and E-cad KO cells expressing LifeAct-GFP were seeded on a glass-bottom imaging dish for live-cell imaging. Time-lapse videos were acquired using temperature (37 °C) and CO_2_ control. Unless otherwise stated, all images or *Z* stacks were acquired in the Airyscan mode without further averaging, and an automated deconvolution was performed within the microscope software (ZEISS ZEN Blue v. 3.1). All the images were visualized using Fiji^62^, and the brightness and contrast were adjusted. For *Z* stacks, the maximum intensity projections or side views were generated.

### Image analysis

#### Approximation of cell–cell adhesion strength

Mixed cultures of MDCK WT and E-cad KO cells were stained for E-cad, α-catenin and β-catenin. To estimate the amount of recruited protein as an approximation of cell–cell adhesion strength, multiple line plots (length, 10 µm; width, 40 pixels) were acquired within the same image. The lines were manually placed perpendicular to and centred on junctions (WT–WT junction, E-cad KO–E-cad KO junction and WT–E-cad KO junction). The line plots were averaged and normalized to the highest average value.

#### Quantification of focal adhesions

Images of paxillin and LifeAct-EGFP (E-cad KO) were acquired at 4,096 × 4,096 pixels Airyscan resolution and averaged four times. The LifeAct-EGFP signal was smoothed first using a 2 × 2 median filter and then using a 10 × 10 median filter. It was manually thresholded to generate a binary image of the E-cad KO cells. A random forest classifier was trained using the pixel classification workflow in ilastik (v. 1.4)^63^ to automatically segment focal adhesions based on the paxillin signal. Using the binary E-cad KO image, the focal adhesion segmentation was split into WT and E-cad KO, resulting in two separate binary images of WT and E-cad KO focal adhesions. In ImageJ, the ‘analyze particles’ function was used to quantify the focal adhesion area and to fit an ellipse to the focal adhesion segmentation. The major axis of the ellipse was used as a measure of the focal adhesion length.

#### Quantification of phospho-myosin intensity

Images of phospho-myosin and LifeAct-EGFP were acquired. Line plots spanning over 100 µm were acquired with the middle of the line placed perpendicular on the cluster edge. These plots were normalized to the maximum intensity value and averaged.

#### Quantification of cell height after tissue collision

Large *Z* stacks of actin and LifeAct-EGFP spanning the interface and the bulk regions of both cell types were acquired. The interface was extracted based on the LifeAct-EGFP signal. Side views of the actin channel were produced and binarized to obtain the cell height. The height was measured continuously over 400 µm with the tissue interface in the middle. Several positions were averaged.

#### Cell segmentation and cell density quantification

Large images of the co-culture stained for ZO1, LifeAct-EGFP (E-cad KO), pHH3 and the nuclei were acquired. Nuclei were segmented using StarDist (v. 0.9*)*^64^ at the default settings and counted using Fiji (the analyze particles function) to calculate the cell densities. pHH3-positive cells were counted manually and their fraction was calculated based on the nuclei segmentation. Cells outlines were segmented based on the ZO1, Nuclei and LifeAct-EGFP signals using Cellpose (v. 2.3)^65^.

#### Interface convexity quantification

Large island of E-cad KO cells within a confluent mixture of KO–WT cells had their interfaces manually segmented using the LifeAct-GFP channel, before and after adding blebbistatin. Convexity was calculated as the ratio between the perimeter of a given island and the perimeter of the corresponding convex shape (smallest polygon that can contain the shape of the island). Computation of the convex bounding region was done using MATLAB’s image processing toolbox (Matlab R2021b).

#### Area fraction quantification

The LifeAct-EGFP signal in the time-lapse videos of the confluent co-culture or collision experiment was converted to 8-bit greyscale and blurred first using a 2 × 2 median filter followed by a 5 × 5 median filter. A binary image was generated using manual thresholding. The binary image was blurred using a 5 × 5 median filter. The intensity of the whole image was measured at all the time points. A 1-h rolling average was applied to compensate for intensity fluctuations in the fluorescent lamp. The area was normalized to the starting value and plotted through time. The absolute area occupied by WT or E-cad KO cells was calculated by multiplying the fraction of E-cad KO cells (intensity divided by 255) with the area in the field of view (0.514188 mm^2^ for the ×10 phase-contrast objective).

#### Extrusion rate quantification

Time-lapse videos (phase-contrast and LifeAct-EGFP signals of E-cad KO cells) of the co-culture or collision experiment were merged. A random forest classifier was trained by manual ground-truth annotation using the pixel classification workflow in ilastik to automatically segment the WT extrusions (extrusion in phase contrast without a LifeAct-EGFP signal) and E-cad KO extrusions (extrusion in phase contrast with a LifeAct-EGFP signal). Cell extrusions result in a strong increase in the phase-contrast signal, with extruded cells appearing as bright spheres. The classifier was trained to detect these bright spheres and separate spheres close to each other, considering not only their brightness but also their roundness and smoothness of their edges. The rest of the image was annotated as the background, particularly cell divisions. When extruded cells die and fragment, they loose these features and were not considered any more (that is, annotated as the background). Cellular identities were attributed based on the LifeAct-EGFP signal. The classification resulted in a three-intensity image (WT extrusion, E-cad KO extrusion and background).

To assess the accuracy of the classifier, we calculated sensitivity (true-positive rate) and specificity (true-negative rate) for WT and E-cad KO extrusions:

Sensitivity: WT (100%), E-cad KO (92 ± 7%)

Specificity: WT (88 ± 5%), E-cad KO (100%)

This means that WT cells are slightly oversegmented and very few E-cad KO cells are wrongly detected as WT, most probably due to inhomogeneities in the LifeAct-EGFP expression strength. For the quantification of extrusion rates described below, this means that E-cad KO rates can be slightly underestimated and WT rates can be slightly overestimated. Due to decreasing performance with strongly increasing extrusion number (objects cannot be separated any more), the analysis was limited to 24 h.

The output was smoothed using a 2 × 2 median filter two times. In ImageJ, TrackMate (v. 7.1)^66^ was used to detect and track extrusions, which were distinguished between WT and E-cad KO based on their intensity. A minimal area threshold (70 pixels) was set to exclude the wrongly detected objects—too small for being a cell. To track extrusions through time and space, a simple linear assignment problem tracker was used with a gap closing distance of 40 pixels and a maximum linking distance of 40 pixels. The minimal length of a track was set to 3, corresponding to 45 min to exclude wrongly detected objects like floating debris or cell divisions. In the resulting output file, each track represents an extrusion event. The time stamp of the first spot defines the extrusion time point and its coordinates define the extrusion position.

To calculate the extrusion rate, the total number of WT or E-cad KO extrusions within 2-h intervals were counted and divided by the area occupied by each cell type. Three consecutive time intervals were averaged, divided by two and normalized to achieve the number of extrusions per hour per square millimetre.

#### Spatial analysis of extrusion events

Using the ImageJ plug-in MorphoLibJ (v. 1.6)^13^ and binary images of the mixed culture, Euclidian distance maps were generated. In these distance maps, for a given position in the image, a pixel has a value equal to the distance to the closest interface between the two cell populations. For each extrusion position, a distance from the interface was associated. Then, using the random probability associated with any given distance as a normalization parameter, the probability distribution of being extruded knowing the distance from the interface was calculated for each cell type composing the mixture of the co-cultures.

#### Traction forces and stress measurements

The bead images obtained during TFM manipulation were merged with the corresponding reference bead images taken after sodium dodecyl sulfate treatment. The resulting stack of images was preprocessed using the Image Stabilizer plug-in in ImageJ^14^ and the illumination was corrected to remove background noise. Displacement field of beads was obtained using PIVlab (v. 3.08)^15^, a particle image velocimetry toolbox developed in MATLAB, with an interrogation window of 32 × 32 pixels and an overlap of 50%. Bead displacements were then correlated to a traction force field using Fourier transform traction cytometry, a known theoretical substrate stiffness and a regularization parameter of 9 × 10^−9^. From the traction force field, we were able to infer the stress tensor everywhere in the tissue using BISM^43^ with a regularization parameter of *Λ* = 10^−6^. Isotropic stress was calculated as half the trace of the stress tensor. To generate the heat map of isotropic stress and traction force magnitude, smoothing was applied through linear interpolation.

#### Stress inference from cell shape

Results computed using BISM were also verified using the method based on the cell shape described in another work^45^. Cell segmentation was done using Cellpose (v. 2.3) (running in Python v. 3) on ZO-1 staining, and the cell identities were attributed according to E-cad KO LifeAct fluorescence.

#### Local characterization of stress around extrusions and cell elimination stress map generation

Using a tuneable square interrogation window, the isotropic stress around each extrusion was extracted 2 h before extrusion until 1 h after extrusion. Extrusion positions were then filtered based on their distance from the interface, depending on the cell type, more or less than 30 µm. From all the remaining extrusions after filtering, a median field of isotropic stress was computed, using which the mean stress evolution or the mean stress fluctuation evolution was plotted. Using the same processed data, cell elimination stress maps were also generated for a moment in time between 40 min and 30 min before extrusion.

#### Calculation of fluctuations

Fluctuation of any parameter (isotropic stress, traction force or bead displacement) was characterized using the susceptibility *χ* (ref. ^52^) and used in other experimental analysis^53^. Briefly, for a given physical parameter *A* distributed inside a population of *N* cells, the susceptibility can be computed as *χ*_*A*_ = Var(*A*) × *N*. In some cases where the number of cells was not convenient to access, the number of pixels in the considered area was used as a proxy to compute the susceptibility, given that all the cells shared the same average area.

### Statistics and reproducibility

All plots/graphs show the mean. All error bars show the standard deviation. All statistical tests were performed using GraphPad Prism (v. 9.5.0), which reports *P* values up to four decimal places. Most representative images correspond to quantifications, representing the number of independent experiments reported there. Unless otherwise stated, all images are representative of at least *N* = 2 independent experiments.

### Computational model

#### 3D phase-field model

We use a recently developed 3D phase-field model for active cell layers^50^. Within this framework, cells are represented as 3D deformable particles that dynamically adapt their shape in response to active stresses as well as interaction forces with other cells and the underlying substrate. In this vein, we consider a cellular monolayer consisting of *N*_cell_ cells on a rigid substrate with its surface normal ${\vec{e}}_{n}\left(={\vec{e}}_{z}\right)={\vec{e}}_{x}\times {\vec{e}}_{y}$ and periodic boundaries in both ${\vec{e}}_{x}$ and ${\vec{e}}_{y}$, where ${\vec{e}}_{x},{\vec{e}}_{y}\,{\rm{and}}\,{\vec{e}}_{z}$ constitute a global orthonormal basis. Each cell *i* is represented by a 3D phase field ${\phi }_{i}={\phi }_{i}(\vec{x},t)$ and initialized with radius *R*_0_. The dynamics associated with the relaxation of the cell interface follows a time-dependent Ginzburg–Landau model with an extra advective term:

1 $${\partial }_{t}{\phi }_{i}+{\vec{v}}_{{\rm{i}}}\cdot \vec{\nabla }{\phi }_{i}=-\varGamma \frac{\delta F}{\delta {\phi }_{i}},\,i=1\ldots{N}_{{\rm{cell}}},$$

where *Γ* is the mobility coefficient. Furthermore, the advective term ${\vec{v}}_{i}\cdot \vec{\nabla }{\phi }_{i}$ updates the location of ${\phi }_{i}={\phi }_{i}(\vec{x},t)$ for each time step and each cell *i* with velocity ${\vec{v}}_{i}$. The free energy functional reads^20^

2 $$\begin{array}{l}F\,=\,\mathop{\sum }\limits_{i}^{{N}_{{{\rm{cell}}}}}\frac{{\gamma }^{i}}{\lambda }\int {\rm{d}}\vec{x}\left\{4{\phi }_{i}^{2}{\left(1-{\phi }_{i}\right)}^{2}+{\lambda }^{2}{\left(\vec{\nabla }{\phi }_{i}\right)}^{2}\right\}\\\qquad+\mathop{\sum }\limits_{i}^{{N}_{{{\rm{cell}}}}}\mu {\left(1-\frac{1}{{V}_{0}}\int {\rm{d}}\vec{x}{\phi }_{i}^{2}\right)}^{2}+\mathop{\sum }\limits_{i}^{{N}_{{{\rm{cell}}}}}\sum _{j\ne i}\frac{{\kappa }_{{{\rm{cc}}}}}{{\lambda }^{2}}\int {\rm{d}}\vec{x}{\phi }_{i}^{2}{\phi }_{j}^{2}\\\qquad+\mathop{\sum }\limits_{i}^{{N}_{{{\rm{cell}}}}}\sum _{j\ne i}{\omega }_{{{\rm{cc}}}}^{i}\int {\rm{d}}\vec{x}\left(\vec{\nabla }{\phi }_{i}\cdot \vec{\nabla }{\phi }_{j}\right)++\mathop{\sum }\limits_{i}^{{N}_{{{\rm{cell}}}}}\frac{{\kappa }_{{{\rm{cs}}}}}{{\lambda }^{2}}\int {\rm{d}}\vec{x}{\phi }_{i}^{2}{\phi }_{w}^{2}\\\qquad\qquad+\mathop{\sum }\limits_{i}^{N}{\omega }_{{{\rm{cs}}}}^{i}\int {\rm{d}}\vec{x}\left(\vec{\nabla }{\phi }_{i}\cdot \vec{\nabla }{\phi }_{w}\right)\end{array}.$$

As such, the free energy stabilizes the cell interface and includes the mechanical properties of the cells such as the cell cortex tension (*γ*^*i*^), as well as gradient contributions ($\vec{\nabla }{\phi }_{i}$) that account for—and distinguish between—cell–cell (${\omega }_{{{\rm{cc}}}}^{i}$) and cell–substrate (${\omega }_{{{\rm{cs}}}}^{i}$) adhesions. In addition to the cortex tension and adhesion terms, compressibility (*µ*) puts a soft constraint on the cell around ${V}_{0}=\left(4/3\right)\uppi {R}_{0}^{3}$ and *κ* captures the repulsion between cell–cell (subscript cc) and cell–substrate (subscript cs) adhesions; *ϕ*_w_ denotes a static phase field representing the substrate (Supplementary Fig. 8 shows the schematic). On the basis of this free energy functional, the interior and exterior of cell *i* corresponds to *ϕ*_*i*_ = 1 and *ϕ*_*i*_ = 0, respectively, connected by a diffuse interface parameterized by length *λ*. To resolve the forces generated at the cellular interfaces, we utilize an overdamped dynamics:

3 $$\vec{{T}_{i}}=\xi \vec{{v}_{i}}-{\vec{F}}_{i}^{\,{\rm{sp}}}=-\int {\rm{d}}\vec{x}\left({{\bf{\Pi }}}^{\mathrm{int}}\cdot \vec{\nabla }{\phi }_{i}\right),$$

where $\vec{{T}_{i}}$ denotes traction as defined for BISM^43^, *ξ* is the substrate friction and ${\vec{F}}_{i}^{\,{\rm{sp}}}=\alpha \vec{{p}_{i}}$ represents self-propulsion forces due to polarity, constantly pushing the system out of equilibrium. In this vein, *α* characterizes the strength of polarity force and

4 $${{\bf{\Pi }}}^{\mathrm{int}}=\left(\mathop{\sum }\limits_{i}^{{N}_{{{\rm{cell}}}}}-\left(\frac{\delta F}{\delta {\phi }_{i}}\right)\right){\bf{1}}+\left(\mathop{\sum }\limits_{i}^{{N}_{{{\rm{cell}}}}}-\left({\zeta }_{{\rm{S}}}^{\,i}{\phi }_{i}{{\bf{S}}}_{i}\right)\right)+\left(\mathop{\sum }\limits_{i}^{{N}_{{{\rm{cell}}}}}-\left({\zeta }_{Q}^{\,i}{\phi }_{{\rm{w}}}{{\bf{Q}}}_{{\rm{w}}}\right)\right),$$

where ${{\bf{S}}}_{i}=\scriptstyle-\int {\rm{d}}\vec{x}\left\{{\left(\vec{\nabla }{\phi }_{i}\right)}^{{\rm{T}}}\vec{\nabla }{\phi }_{i}\right\}+\left(\frac{1}{3}\right){{\rm{Tr}}}\int {\rm{d}}\vec{x}\left\{{\left(\vec{\nabla }{\phi }_{i}\right)}^{{\rm{T}}}\vec{\nabla }{\phi }_{i}\right\}$ and ${{\bf{Q}}}_{w}=\left(\frac{d}{d-1}\right)\left(\vec{n}\otimes \vec{n}-\frac{1}{d}{\left(\vec{n}\right)}^{2}{\bf{1}}\right)$ (*d* = 3 is the dimension); ${\zeta }_{Q}^{\,i}$ and ${\zeta }_{S}^{\,i}$ are the strength of cell–substrate and cell–cell active stresses, respectively; and **I** is the identity tensor. In the model, $\vec{n}$ represents the orientation of the stress fibres of the substrate, for example, $\vec{n}=\left(\cos \upsilon ,\sin \upsilon ,0\right)$ and *υ* = 0 on the surface of the substrate. Furthermore, the dynamics of cell polarity is introduced based on the contact inhibition of locomotion^67,68^ by aligning the polarity of the cell to the direction of the total interaction force acting on the cell^24^. As such, the polarization dynamics is given by

$${\partial }_{t}{\theta }_{i}=-\frac{1}{{\tau }_{{{\rm{pol}}}}}\Delta {\varTheta }_{i}+{D}_{{\rm{r}}}\eta (t),$$

where *θ*_*i*_ ∈ [–π, π] is the angle associated with the polarity vector $\vec{{p}_{i}}=\left(\cos {\theta }_{i},\sin {\theta }_{i},0\right)$ and *η*(*t*) is a standard Gaussian white noise with zero mean unit variance, *D*_r_ is the rotational diffusivity, Δ*θ*_*i*_ is the angle between $\vec{{p}_{i}}$ and $\vec{{T}_{i}}$, and positive constant *τ*_pol_ sets the alignment timescale. Finally, we compute a coarse-grained stress field ${{\bf{\sigma }}}^{i}={{\bf{\sigma }}}^{i}(\vec{x},t)$ that encodes both active and passive contributions on a discretized domain for node *i* as

$${{\bf{\sigma }}}^{i}=\frac{1}{{a}_{0}^{3}}\mathop{\sum }\limits_{j}^{{N}_{i}}{\vec{r}}^{{ij}}\otimes {\vec{T}}^{\,j},$$

where *a*_0_ = 1 is the grid size and corresponds to spatial-domain discretization, ${\vec{r}}^{{ij}}=({\vec{x}}^{i}-{\vec{x}}^{j})$ and *N*_*i*_ is the number of nearest neighbours at node *i*. A negative stress value indicates compression and a positive value, tension.

### 3D phase-field model simulation details

Specifically, we consider a cellular monolayer consisting of *N* = 400 cells on a rigid substrate. Cells are initiated on a two-dimensional simple cubic lattice and inside a cuboid of size *L*_*x*_ = *L*_*y*_ = 320, *L*_z _= 64 and radius *R*_0_ = 8. The total number of time steps in the simulations are *n*_sim_ = 15,000.

Unless specified otherwise, time $\widetilde{t}=t/\tau$, where $\tau =\left(2{R}_{0}\right)/\bar{v}$, $\bar{v}\left(=0.02\right)$ in the simulation units is the average speed of cells and *τ* represents the characteristic time for a cell to move a distance equivalent to its size. With this normalization, the typical MDCK cell speed is ~20 µm h^–1^ and cell size is ~20 µm. The physical properties are as follows unless specified otherwise: *γ* = 0.008, *μ* = 45, *ξ* = 1, *ζ*_S_ = 4 × 10^–5^ and *ζ*_Q_ = –0.01.

### Energetics model

The energetics model is based on the work done to extrude the cell. There are two contributions to the work: adhesion and surface tension. The work needed to increase the surface area by d*A* against surface tension is given by *k*d*A*_tot_. By contrast, for adhesion, the cell is doing work to increase the surface area. Therefore, the total work is

5 $${{\rm{d}}W}=-{\omega }_{i}{\rm{d}}{A}_{i}+{k{\rm{d}}}{A}_{{{\rm{tot}}}}.$$

The subscript *i* refers to cell–cell adhesion and cell–substrate adhesion. The negative sign in front of *ω*_*i*_ indicates that the cell is doing the adhesive work. Consider the various contributions to the adhesion energy for cell type I. There is the cell–substrate adhesion energy of cell type I. For cell type II, cell–cell adhesion energy of cell type I with both type-I and type-II neighbours. Each energy is proportional to the change in the respective contact area. Expanding,

6 $${\rm{d}}{W}^{\,{\rm{I}}}=-{\omega }_{{\rm{s}}}^{{\rm{I}}}{\rm{d}}{A}_{{\rm{s}}}^{{\rm{I}}}-{\omega }_{{\rm{c}}}^{{\rm{I}}}{\rm{d}}{A}_{{{\rm{cc}}}}^{{\rm{I}}}-{\omega }_{{\rm{c}}}^{{\rm{I}}-{{\rm{II}}}}{\rm{d}}{A}_{{{\rm{cc}}}}^{{\rm{I}}-{{\rm{II}}}}+{\omega }_{{\rm{s}}}^{{{\rm{neigh}}}}{\rm{d}}{A}_{{\rm{s}}}^{{{\rm{neigh}}}}+{k}^{{\rm{I}}}{\rm{d}}{A}_{{{\rm{tot}}}}^{{\rm{I}}}.$$

The first term represents the cell–substrate adhesion of the cell at the interface. The second term represents the cell–cell adhesion of the cell with neighbours of the same type. The third term is the cell–cell adhesion with a neighbour of the other type. When the cell is extruded, the substrate area it used to occupy is occupied by its neighbours. Therefore, there is an additional energy—the fourth term—from the substrate energy of the neighbours. The last term is the intrinsic stiffness of the cell.

Half the cell is assumed to be in contact with the cell of the same type and half with the other type. A factor of 0.5 is chosen for simplicity. We assume that the winning cell pushes the losing cell from the bottom and, hence, occupies the entire substrate contact area. The energy difference d*W*^II^ – d*W*^I^ decides which of the cell types wins at an interface. Notice that the ${\omega }_{{\rm{c}}}^{{\rm{I}}-{{\rm{II}}}}$ term will be the same in both work functions d*W*^I^ and d*W*^II^. It will, therefore, be dropped. Now, the work done is

7 $${\rm{d}}{W}^{\,{\rm{I}}}=\left({\omega }_{{\rm{s}}}^{{{\rm{II}}}}-{\omega }_{{\rm{s}}}^{{\rm{I}}}\right){\rm{d}}{A}_{{\rm{s}}}^{{\rm{I}}}-{\omega }_{{\rm{c}}}^{{\rm{I}}}{\rm{d}}{A}_{{{\rm{cc}}}}^{{\rm{I}}}+{k}^{{\rm{I}}}{\rm{d}}{A}_{{{\rm{tot}}}}^{{\rm{I}}}.$$

The change in area is directly calculated using the surface areas of the shapes shown in the schematic (Supplementary Fig. 7), in which each shape is assumed to have the same volume. The cell is initially assumed to be a cylinder, whose basal radius is allowed to vary. Stress fluctuations lead to the shrinking of the basal radius, leading to a cone. The work needed to break the cell–cell adhesion bond is calculated by changing the shape of the cell from a cone to a sphere, again with a constant volume. The radius of the sphere and the apical radius are both assumed to be constant at 10 µm. Therefore, the volume is $\frac{4}{3}\uppi \times{10}^{3} \approx \,4,188.8\,\upmu {{\rm{{m}}^{3}}}$. The difference in work (Fig. 2g) is normalized by the quantity ${\rm{d}}{W}_{0}^{k}$. This is the energy released by a cell to go through the process if there was no cell–cell or cell–substrate adhesion.

#### Ethics statement

The study was approved by the local ethics committee (Breast Group of René Huguenin Hospital, Saint-Cloud, France).

### Reporting summary

Further information on research design is available in the Nature Portfolio Reporting Summary linked to this article.

## Online content

Any methods, additional references, Nature Portfolio reporting summaries, source data, extended data, supplementary information, acknowledgements, peer review information; details of author contributions and competing interests; and statements of data and code availability are available at 10.1038/s41563-025-02150-9.

## Supplementary information

Supplementary Information Supplementary Figs. 1–10 and Captions for Supplementary Videos 1–9.

Reporting Summary

Supplementary Video 1 **Time-lapse video of competition within patient-derived metaplastic cancer cells**. Phase-contrast images of E-cad^+^ cells surrounded by E-cad^−^ cells, as shown in Fig. 1b. Frame rate, 10 min. Scale bar, 200 µm.

Supplementary Video 2 **Time-lapse video of competition within patient-derived metaplastic cancer cells derived from the second patient**. Phase-contrast images of E-cad^+^ cells surrounded by E-cad^−^ cells corresponding to Supplementary Fig. 1c. Frame rate, 10 min. Scale bar, 200 µm.

Supplementary Video 3 **Time-lapse video of competition between MDCK WT and MDCK E-cad KO cells**. Phase-contrast images of E-cad KO cells expressing LifeAct-EGFP in green. Frame rate, 15 min. Scale bar, 100 µm.

Supplementary Video 4 **Time-lapse video of competition between MDCK dKO and MDCK E-cad KO cells**. Phase-contrast images of E-cad KO cells expressing LifeAct-EGFP in green. Frame rate, 15 min. Scale bar, 100 µm.

Supplementary Video 5 **Laser ablation of WT cluster within the mixed culture**. WT cells express CAAX-GFP and E-cad KO cells express LifeAct-EGFP. Video shows the data in Supplementary Fig. 5d. The magenta line shows the ablation area. Frame rate, 5 s. Scale bar, 50 µm.

Supplementary Video 6 **Laser ablation of E-cad KO cluster within the mixed culture**. WT cells express CAAX-GFP and E-cad KO cells express LifeAct-EGFP. Video shows the data in Supplementary Fig. 5d. The magenta line shows the ablation area. Frame rate, 5 s. Scale bar, 50 µm.

Supplementary Video 7 **Time-lapse video of cell eliminations at the interface between MDCK WT and MDCK E-cad KO cells**. Phase-contrast images of E-cad KO expressing LifeAct-EGFP in green. The magenta line highlights the interface. Note that the E-cad KO cells are only eliminated when directly in contact with the WT cells. Frame rate, 15 min. Scale bar, 50 µm.

Supplementary Video 8 **Example video showing simulation of competition between mWT and mE-cad KO cells**. mWT cells are shown in blue, and mE-cad KO cells in red. The cell–cell adhesion strength of mWT cells is eight times higher than that in mE-cad KO cells, but the cell–substrate adhesion strength is constant.

Supplementary Video 9 **Time-lapse video of actin dynamics in competition between MDCK WT and E-cad KO cells**. Confocal images showing the maximum projection of actin expressed in both cell types. 60% MDCK WT cells express LifeAct-mRuby (displayed in white) and all E-cad KO cells express LifeAct-EGFP (displayed in green). Note the protrusion activity of E-cad KO cells at the interface. Frame rate, 7.5 min. Scale bar, 20 µm.

Supplementary Data 1 Source data for Supplementary Figs. 2–5.

## Source data

Source Data Fig. 1 Statistical source data.

Source Data Fig. 2 Statistical source data.

Source Data Fig. 3 Statistical source data.

Source Data Fig. 4 Statistical source data.

Source Data Fig. 5 Statistical source data.

Source Data Extended Data Fig. 1 Statistical source data.

Source Data Extended Data Fig. 2 Statistical source data.

Source Data Extended Data Fig. 3 Statistical source data.

Source Data Extended Data Fig. 4 Statistical source data.

Source Data Extended Data Fig. 5 Statistical source data.

Source Data Extended Data Fig. 6 Statistical source data.

Source Data Extended Data Fig. 7 Statistical source data.

Source Data Extended Data Fig. 8 Statistical source data.

Source Data Extended Data Fig. 9 Statistical source data.

Source Data Extended Data Fig. 10 Statistical source data.

## Data Availability

All data supporting the findings of this study are included within the Article and its Supplementary Information. Source data are provided with this paper.

## Code availability

The code for the phase-field simulations is available via GitHub at https://github.com/siavashmonfared/celadro_three_dimensional.
