# Supplementary material for: Force transmission is a master regulator of mechanical cell competition
Source: Nat Mater. 2025 Mar 14;24(6):966–76. doi: 10.1038/s41563-025-02150-9 (PMC12133594; doi:10.1038/s41563-025-02150-9)
Supplement: Supplementary file 1 — Supplementary Figs. 1–10 and Captions for Supplementary Videos 1–9. [file 41563_2025_2150_MOESM1_ESM.pdf]

nature materials

Supplementary information https://doi.org/10.1038/s41563-025-02150-9

Force transmission is a master regulator of mechanical cell competition

In the format provided by the authors and unedited

The PDF file includes:

Supplementary Video captions 1 to 9
Supplementary Figures 1 to 10

Other Supplementary Materials for this manuscript include the following:

Supplementary Videos 1 to 9
Source data files

Video captions:

Video S1: Timelapse video of competition within patient-derived metaplastic cancer cells. Phase contrast images of E-cad$^+$ cells surrounded by E-cad$^-$ cells corresponding to **Figure 1B**. Frame rate 10 min. Scale bar 200 µm.

Video S2: Timelapse video of competition within patient-derived metaplastic cancer cells derived from a second patient. Phase contrast images of E-cad$^+$ cells surrounded by E-cad$^-$ cells corresponding to **Fig. S1 C**. Frame rate 10 min. Scale bar 200 µm.

Video S3: Timelapse video of competition between MDCK WT and MDCK E-cad KO cells. Phase contrast images of E-cad KO cells expressing LifeAct-EGFP in green. Frame rate 15 min. Scale bar 100 µm.

Video S4: Timelapse video of competition between MDCK dKO and MDCK E-cad KO cells. Phase contrast images of E-cad KO cells expressing LifeAct-EGFP in green. Frame rate 15 min. Scale bar 100 µm.

Video S5: Laser ablation of WT cluster within the mixed culture. WT cells express CAAX-GFP and E-cad KO cells express LifeAct-EGFP. Video corresponds to Fig. S5 D. Magenta line shows ablation area. Frame rate 5 sec. Scale bar 50 µm.

Video S6: Laser ablation of E-cad KO cluster within the mixed culture. WT cells express CAAX-GFP and E-cad KO cells express LifeAct-EGFP. Video corresponds to Fig. S5 D. Magenta line shows ablation area. Frame rate 5 sec. Scale bar 50 µm.

Video S7: Timelapse video of cell eliminations at the interface between MDCK WT and MDCK E-cad KO cells. Phase contrast images of E-cad KO expressing LifeAct-EGFP in green. Magenta line highlights the interface. Note that E-cad KO cells are not only eliminated when directly in contact with WT cells. Frame rate 15 min. Scale bar 50 µm.

Video S8: Example video showing simulation of competition between model WT and model E-cad KO cells. mWT cells are shown in blue, mE-cad KO cells in red. The cell-cell adhesion strength of mWT cells is 8x higher than the mE-cad KO, the cell-substrate adhesion strength is constant.

Video S9: Timelapse video of actin dynamics in competition between MDCK WT and E-cad KO cells. Confocal images showing maximum projection of actin expressed in both cell types. 60% MDCK WT cells express LifeAct-mRuby, displayed in white, all E-cad KO cells express LifeAct-EGFP, displayed in green. Note the protrusion activity of E-cad KO cells at the interface. Frame rate 7.5 min. Scale bar 20 µm.

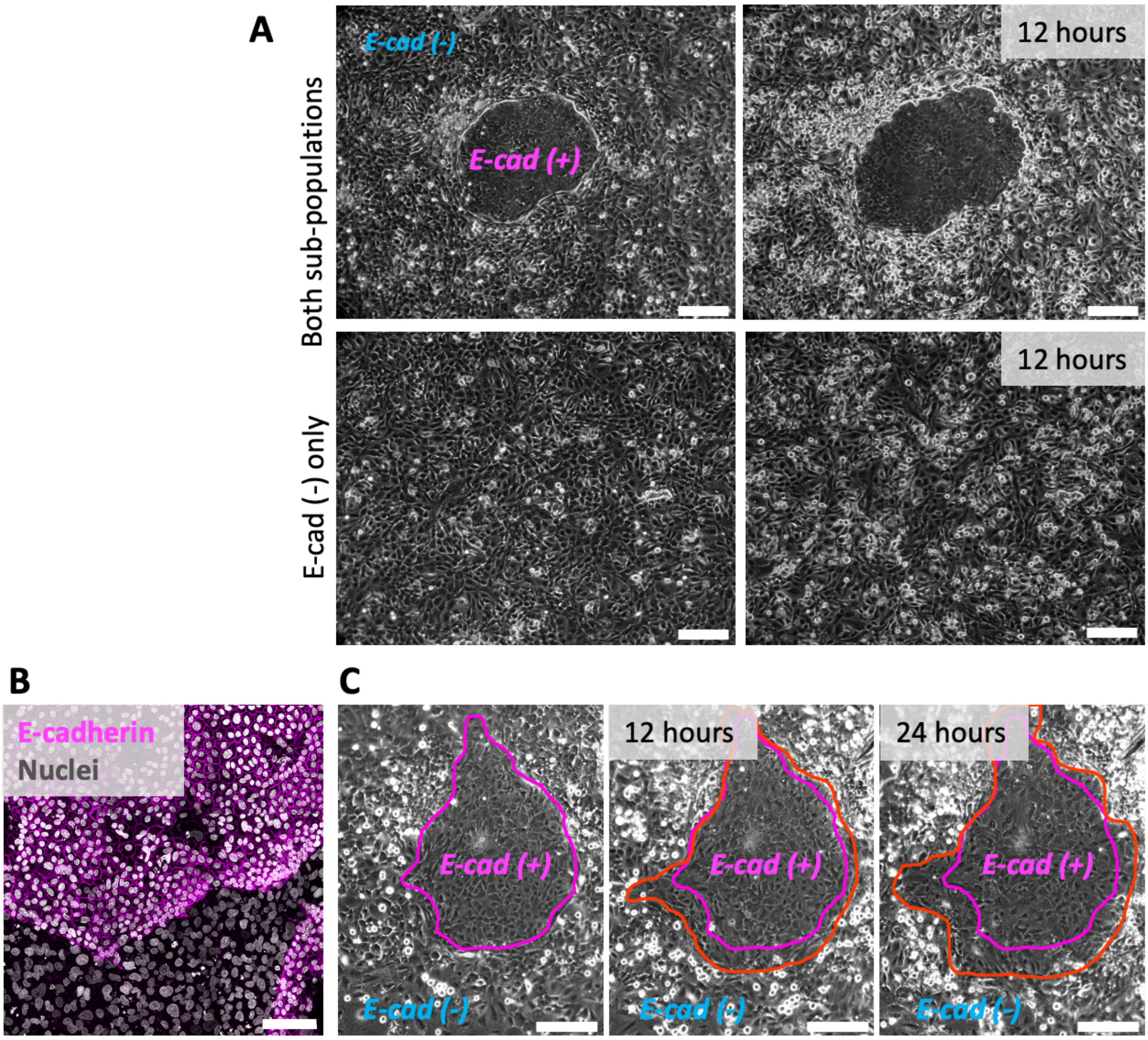

Supplementary Figure 1: E-cad^{-} triple negative tumor cells are viable and sample from second patient (**A**) Upper: Mix of both sub-populations. The E-cad^{+} population is spreading, leading to eliminations of E-cad^{-} cells in its vicinity. Lower: E-cad^{-} cells grow to high densities independent of interactions with E-cad^{+} cells. Over time, elimination at random positions can be observed, but fewer than when interacting with the E-cad^{+} sub-population. (**B**) Confocal image of metaplastic breast cancer cells derived from a second patient stained for E-cadherin (magenta). E-cad^{+} and E-cad^{-} cells have sorted. (**C**) Phase contrast images of cells corresponding to (**B**). Magenta line shows initial boundaries of the sub-populations red lines show boundaries at the given timepoint. Representative images from N=3 independent experiments. Scale bars 200 µm.

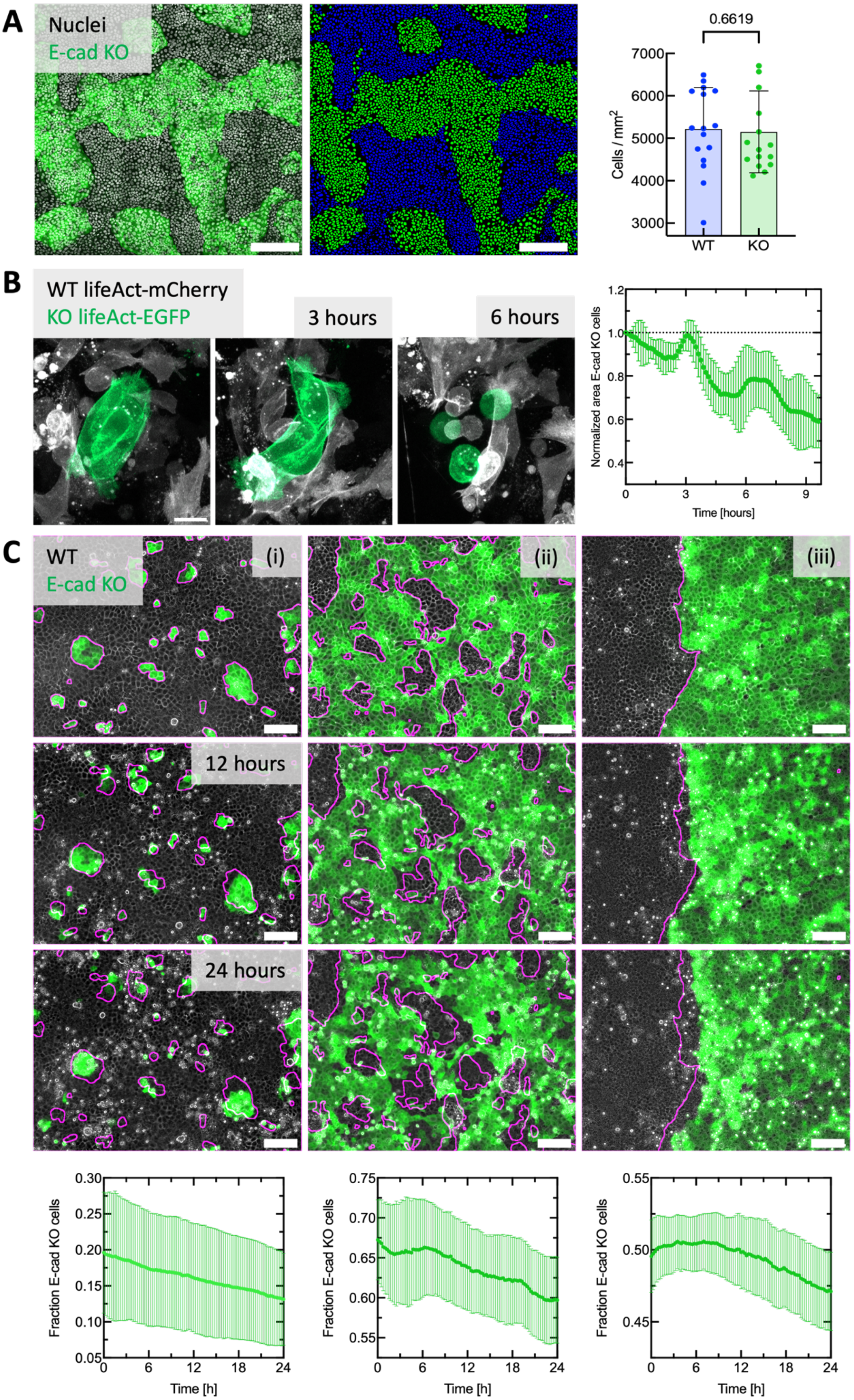
A
Nuclei
E-cad KO
0.6619
Cells / mm²
7000
6000
5000
4000
3000
WT
KO
B
WT lifeAct-mCherry
KO lifeAct-EGFP
3 hours
6 hours
Normalized area E-cad KO cells
1.2
1.0
0.8
0.6
0.4
0
3
6
9
Time [hours]
C
WT
E-cad KO
(i)
(ii)
(iii)
12 hours
24 hours
Fraction E-cad KO cells
0.30
0.25
0.20
0.15
0.10
0.05
0.75
0.70
0.65
0.60
0.55
0.50
0.45
0
6
12
18
24
Time [h]

Supplementary Figure 2: E-cad KO cells lose independent of ratio or level of sorting (**A**) Confocal image of nuclei (white) and E-cad KO cells (green). Middle: Corresponding nuclei segmentation using StarDist. The cellular identity was determined based on the E-cad KO fluorescence. Right: Average cell densities in mixed cultures. Each datapoint shows the cell density for one FOV. n=16 FOV from N=2 independent experiments. P-value from unpaired, two-sided t-test. (**B**) Left: Snapshots from Airyscan confocal movies of WT cells expressing LifeAct-mCherry and E-cad KO cells expressing LifeAct-EGFP. The E-cad KO cells are eliminated by LifeAct expressing WT cells. Right: Area quantifications corresponding to images on the left from n=4 movies from N=1 experiment. (**C**) Brightfield and fluorescence images of WT and E-cad KO cells (green) at the beginning of the experiment and 12 and 24 hours later. Magenta lines show initial cluster sizes to visualize cluster progression over time. Bottom row: Area development of E-cad KO cells. (**i**) E-cad KO in minority. (**ii**) E-cad KO in majority. (**iii**) Collision of E-cad KO and WT tissues. Movies are analysed shortly after contact formation. Area quantifications from (**i**) n=8 movies from N=3 independent experiments, (**ii**) n=7 movies from N=3 independent experiments, (**iii**) n=4 movies from N=2 independent experiments. Data are presented as mean values +/- SD. Scale bars 200 µm (A), 100 µm (C), 10 µm (B).

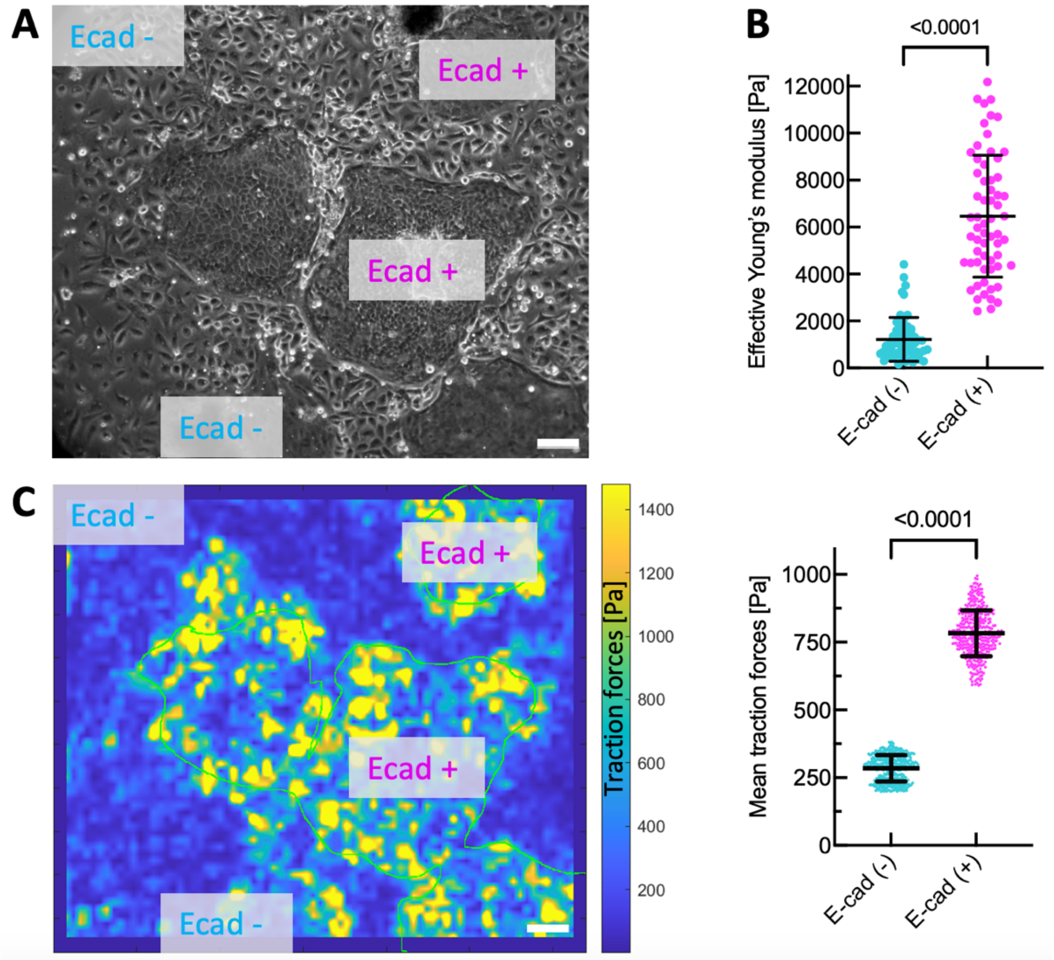

Supplementary Figure 3: Mechanics of intratumoral cell competition (**A**) Brightfield image of E-cad (-) and E-cad (+) tumor clusters, as shown in **Figure 1**. (**B**) Cell stiffnesses measured with nanoindentation. Each datapoint shows one measurement. N=2 independent experiments and n=57 indentations. (**C**) Map of traction forces corresponding to (**A**). Green lines show cluster outlines. Right: Average traction forces. Each datapoint shows the average value from one field-of-view at one timepoint. N=2 independent experiments and n=408 timepoints. P-values from two-sided t-test. Data are presented as mean values +/- SD. Scale bars 100 µm.

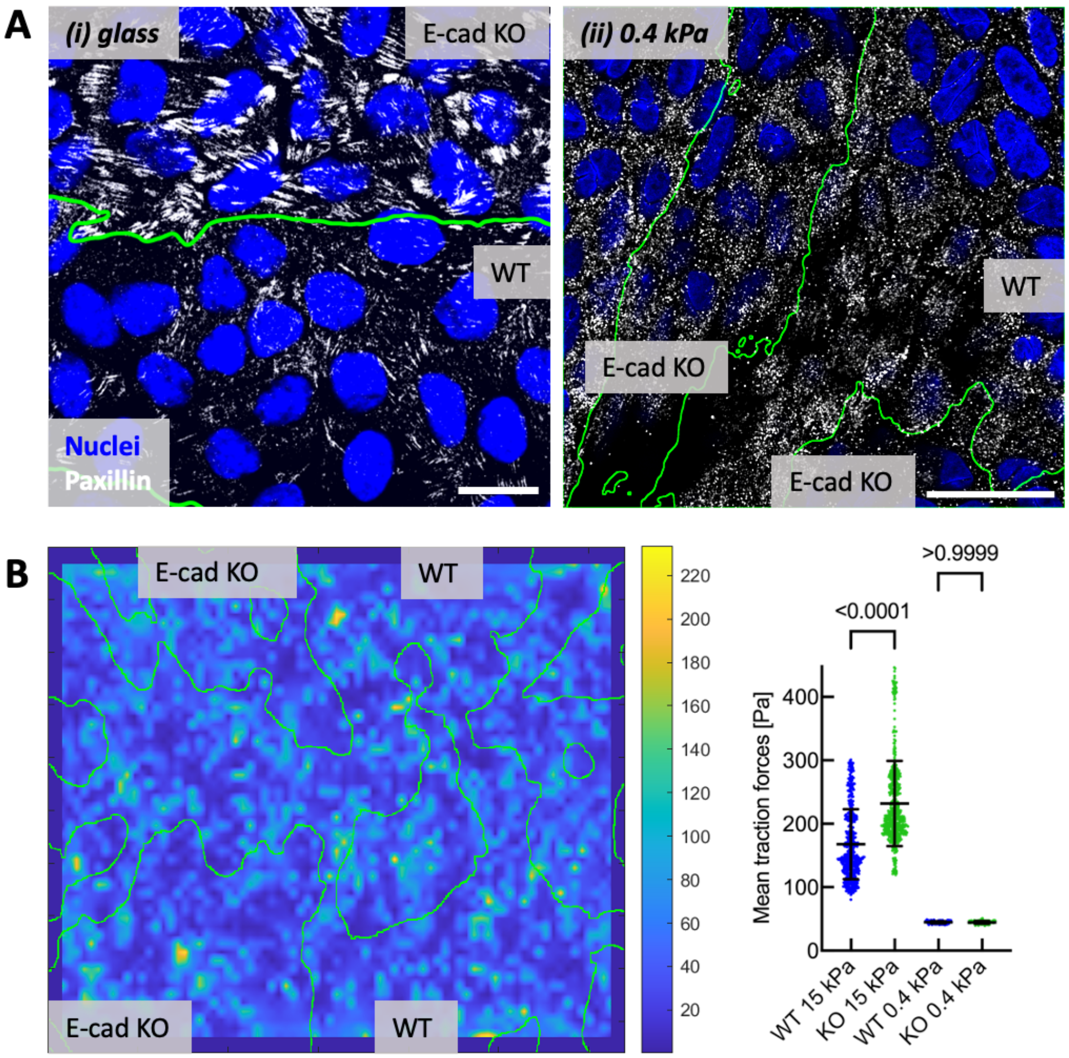

Supplementary Figure 4: Mixed culture with low cell-substrate adhesion (**A**) Confocal images of focal adhesions (Paxillin, white) in mixed culture grown on (i) glass or on (ii) soft substrate (370 Pa, PAA). (**B**) Left: map of traction forces on soft substrate. Right: Average traction forces on different substrate stiffnesses. Green lines show outlines of E-cad KO cluster. Each datapoint represents one average map. n=568 positions from N=4 independent experiments (15 kPa) and n=170, N=2 (370 Pa). P-values from Kruskal-Wallis test corrected for multiple comparisons (Dunn's test). Data are presented as mean values +/- SD. Scale bars 20 µm.

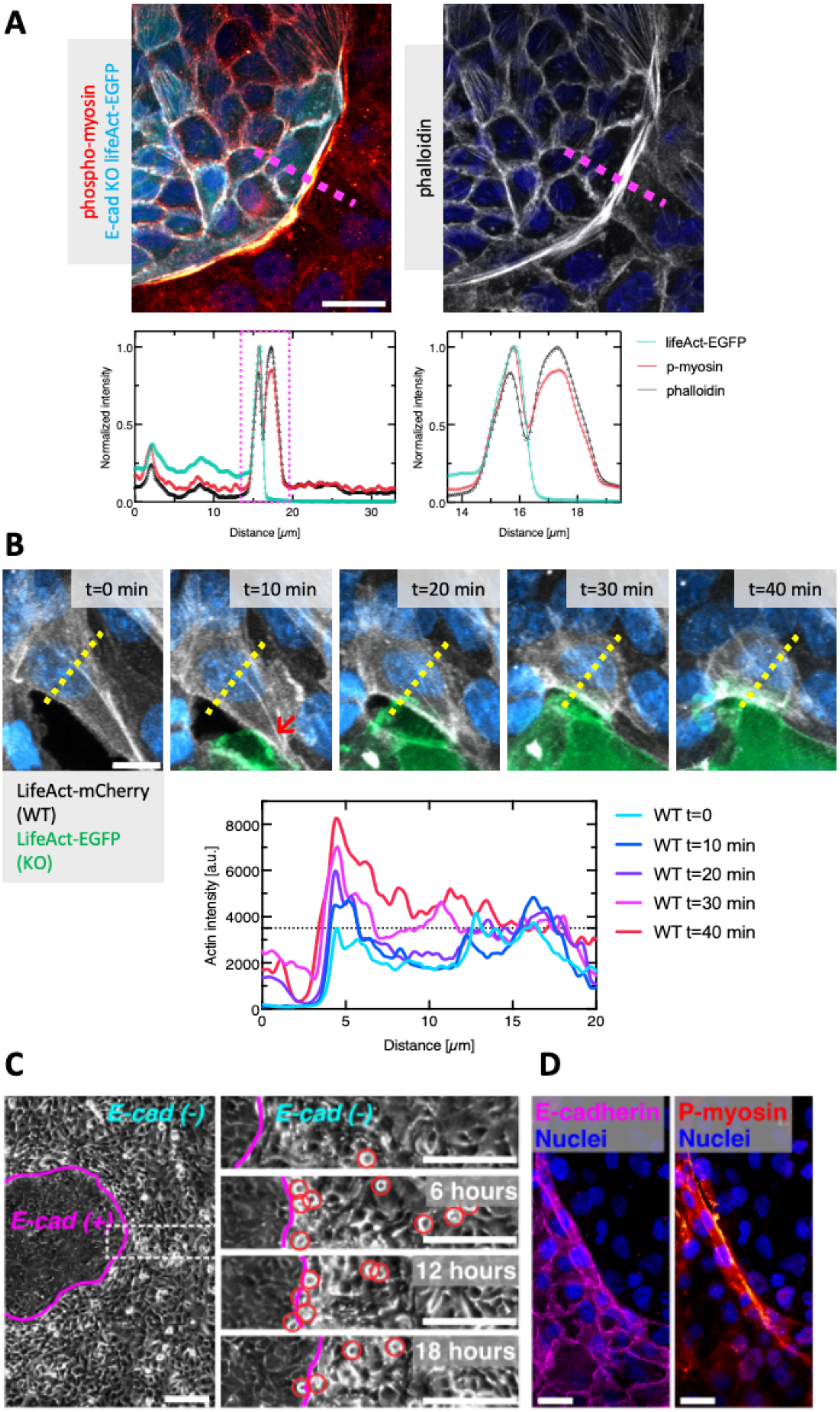
A
phospho-myosin
E-cad KO lifeAct-EGFP
phalloidin
Normalized intensity
Distance [μm]
lifeAct-EGFP
p-myosin
phalloidin
B
t=0 min
t=10 min
t=20 min
t=30 min
t=40 min
LifeAct-mCherry (WT)
LifeAct-EGFP (KO)
Actin intensity [a.u.]
Distance [μm]
WT t=0
WT t=10 min
WT t=20 min
WT t=30 min
WT t=40 min
C
E-cad (-)
E-cad (+)
6 hours
12 hours
18 hours
D
E-cadherin
Nuclei
P-myosin
Nuclei

Supplementary Figure 5: Actomyosin accumulation at interface between E-cad KO and WT cells and interface elimination during intratumoral competition (**A**) Representative airyscan confocal image of increased phospho-myosin accumulating in both cell types at the interface. Intensity line plots were drawn at the indicated position for actin (phalloidin), phospho-myosin and LifeAct-EGFP (E-cad KO cells). Zoom-in on intensity profile reveals two separate intensity peaks in WT and E-cad KO and the colocalization of LifeAct-EGFP and phospho-myosin. (**B**) Actin dynamics following cell-cell collision. WT cells express LifeAct-mCherry (white) and E-cad KO cells express LifeAct-EGFP (green). Intensity values were measured at the indicated line. An increase in actin intensity within the WT cells was observed following collision (red arrow) at new interface with E-cad KO cells. (**C**) Representative phase-contrast image of patient-derived tumor xenograft cultured in 2D. E-cad (-) cells are eliminated at the interface. (**D**) Confocal images showing an increase of phospho-myosin at the tissue interface. Scale bars 200 µm (**C**), 20 µm (**A**), 25 µm (**D**), 10 µm (**B**).

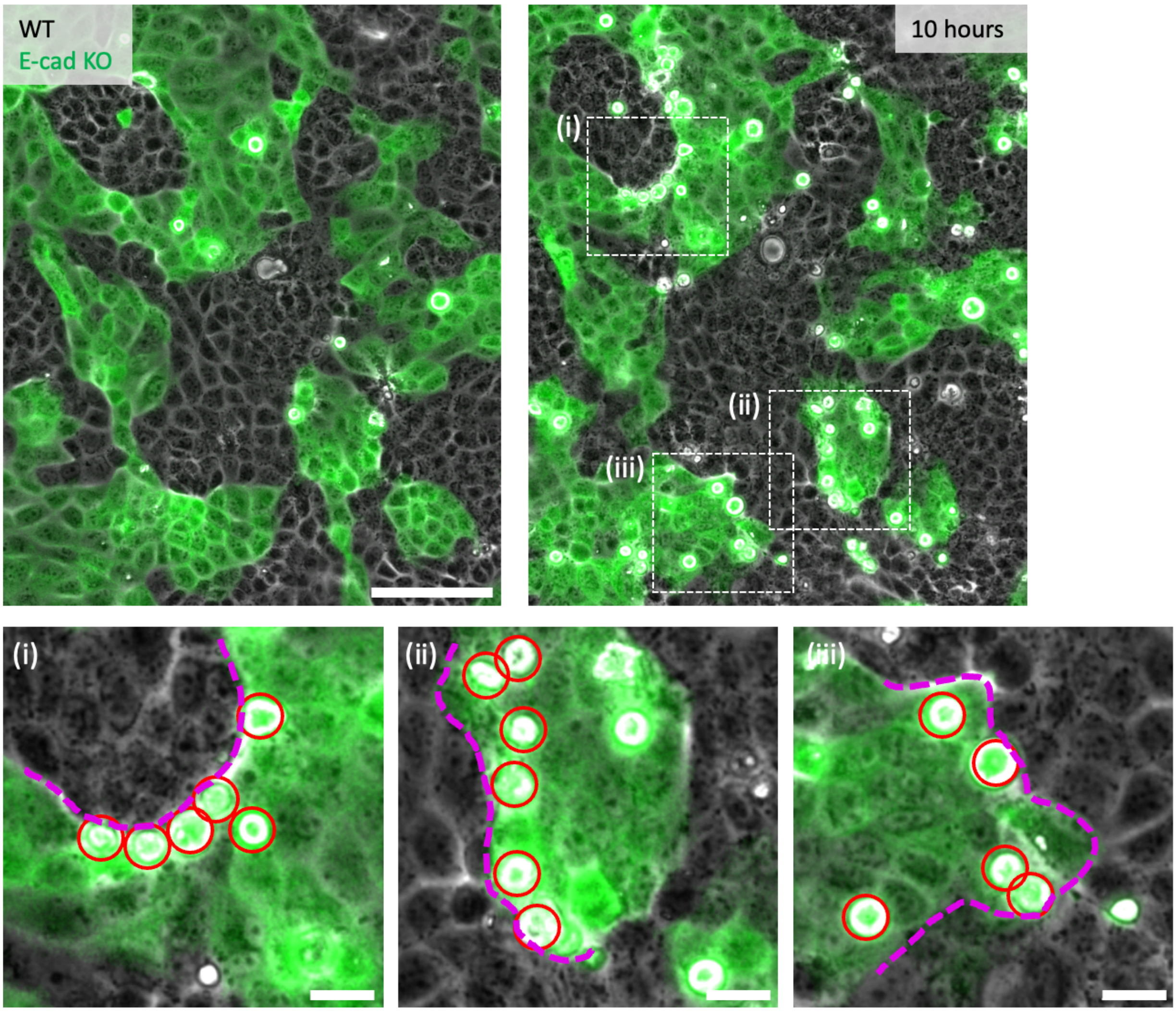

Supplementary Figure 6: Preferred interface elimination is independent of interface curvature Brightfield and fluorescence (E-cad KO) images of a competing MDCK coculture. Within the same field of view, E-cad KO cells are eliminated at positively (i), minimally (ii) or negatively (iii) curved interfaces (dashed lines). Extrusions indicated by red circles. Scale bars: 100 µm (overview), 10 µm (zoom-ins).

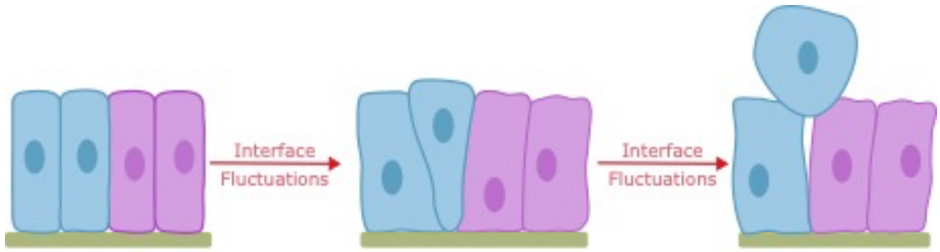

Supplementary Figure 7: Schematic of shape changes The analytical model uses these three shapes: a cylinder, a cone and a sphere, to calculate the change in area used to find the work. All three cell shapes have the same volume.

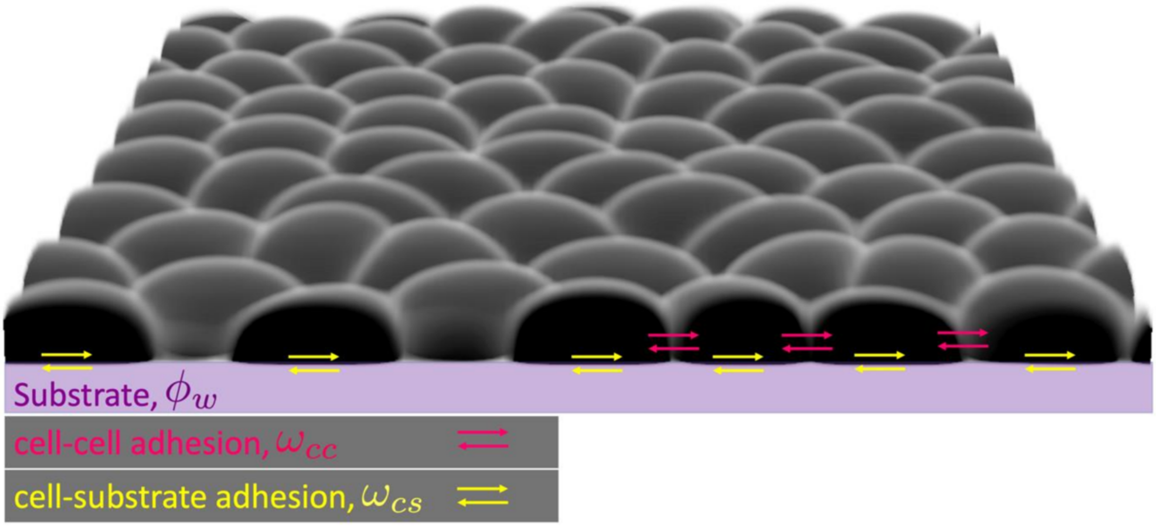

Supplementary Figure 8: Key features of the multi-phase field model An example cross-section from a simulation. The arrows show, schematically, how cell-cell and cell-substrate adhesions are explicitly accounted for in the model and mathematically outlined in the Methods.

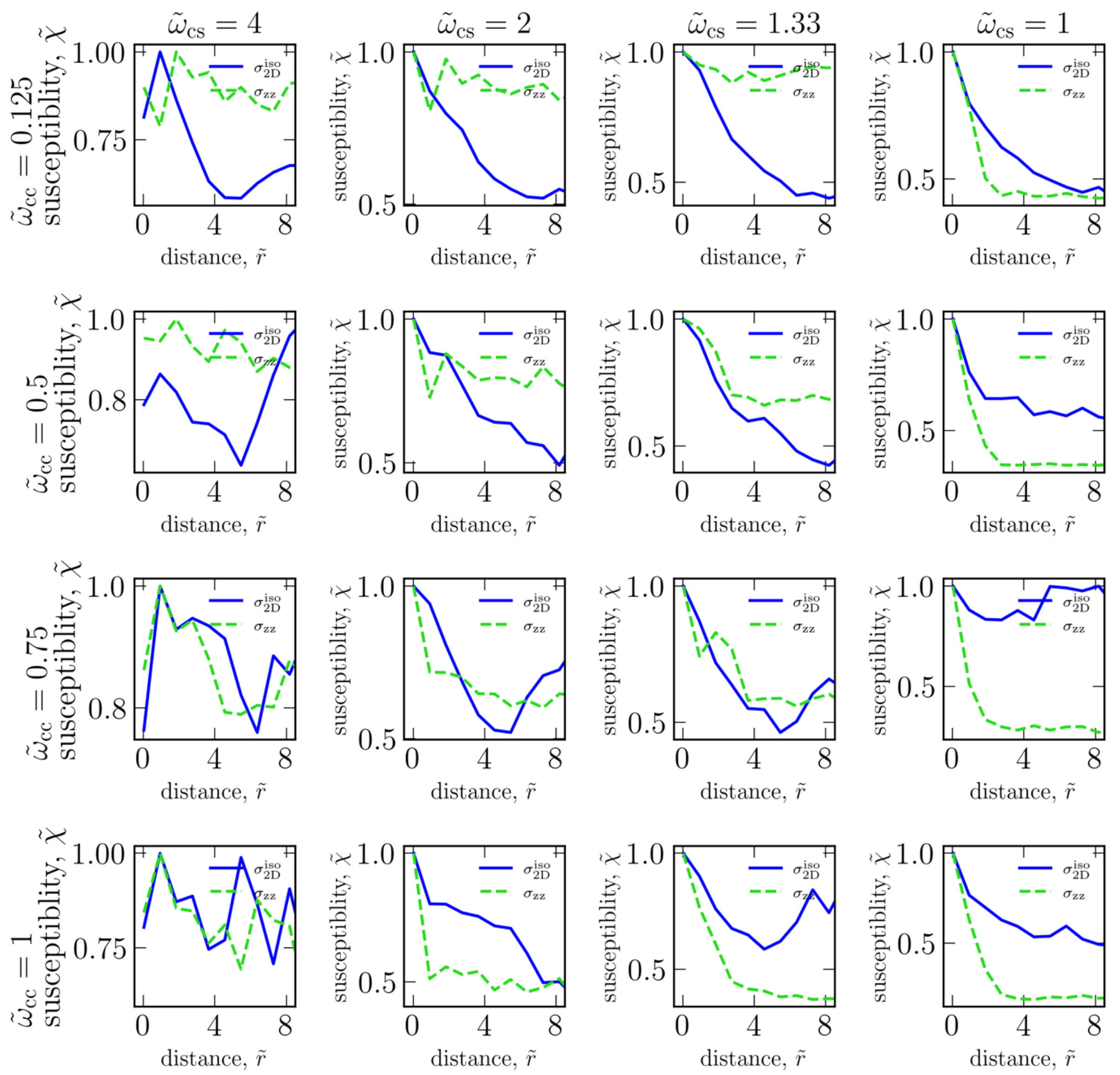

Supplementary Figure 9: Stress fluctuations away from the interface highlight the role of stress transmission Susceptibility of two-dimensional isotropic stress (σ_{2D}^{iso}), and the out-of plane stress component (σ_{zz}) for mE-cad KO cells as a function of distance away from the interface for various cell-substrate adhesion ($\widetilde{\omega}_{cs}$) and cell-cell adhesion ($\widetilde{\omega}_{cc}$) strengths. The distance is normalized by the initialized cell radius. The susceptibility is normalized by the maximum value.

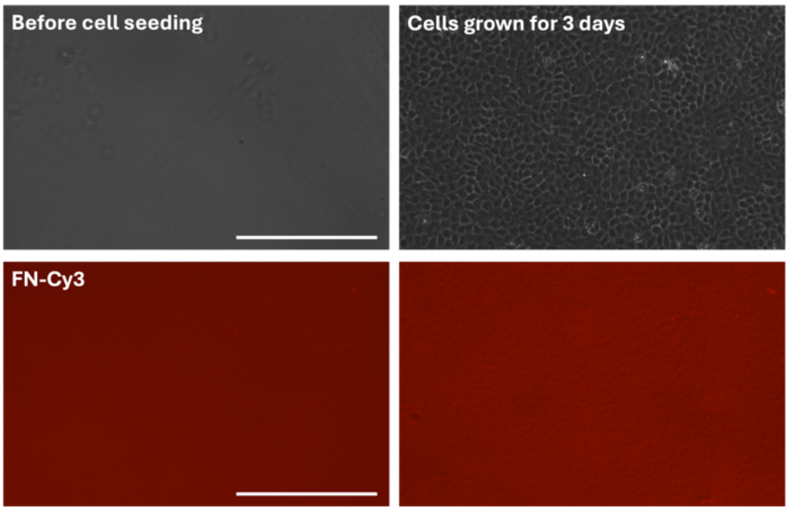

Supplementary Figure 10: Phase contrast and fluorescence images of 15 kPa PDMS coated with Cy3-labelled fibronectin. Left: Before cell seeding, right: after 3 days of cell culture. Scale bars 200 µm.
