# Supplementary material for: Force transmission is a master regulator of mechanical cell competition
Source: Nat Mater. 2025 Mar 14;24(6):966–76. doi: 10.1038/s41563-025-02150-9 (PMC12133594; doi:10.1038/s41563-025-02150-9)
Supplement: Supplementary file 2 — Reporting Summary [file 41563_2025_2150_MOESM2_ESM.pdf]

nature portfolio

Corresponding author(s): Rene Marc Mege
Amin Doostmohammadi
Benoit Ladoux

Last updated by author(s): Jan 5, 2025

Reporting Summary

Nature Portfolio wishes to improve the reproducibility of the work that we publish. This form provides structure for consistency and transparency in reporting. For further information on Nature Portfolio policies, see our Editorial Policies and the Editorial Policy Checklist.

Statistics

For all statistical analyses, confirm that the following items are present in the figure legend, table legend, main text, or Methods section.

n/a	Confirmed	
☐	☒	The exact sample size (*n*) for each experimental group/condition, given as a discrete number and unit of measurement
☐	☒	A statement on whether measurements were taken from distinct samples or whether the same sample was measured repeatedly
☐	☒	The statistical test(s) used AND whether they are one- or two-sided<br>*Only common tests should be described solely by name; describe more complex techniques in the Methods section.*
☒	☐	A description of all covariates tested
☐	☒	A description of any assumptions or corrections, such as tests of normality and adjustment for multiple comparisons
☐	☒	A full description of the statistical parameters including central tendency (e.g. means) or other basic estimates (e.g. regression coefficient) AND variation (e.g. standard deviation) or associated estimates of uncertainty (e.g. confidence intervals)
☐	☒	For null hypothesis testing, the test statistic (e.g. *F*, *t*, *r*) with confidence intervals, effect sizes, degrees of freedom and *P* value noted<br>*Give P values as exact values whenever suitable.*
☒	☐	For Bayesian analysis, information on the choice of priors and Markov chain Monte Carlo settings
☒	☐	For hierarchical and complex designs, identification of the appropriate level for tests and full reporting of outcomes
☒	☐	Estimates of effect sizes (e.g. Cohen's *d*, Pearson's *r*), indicating how they were calculated

Our web collection on statistics for biologists contains articles on many of the points above.

Software and code

Policy information about availability of computer code

Data collection	Imaging data was acquired using a Nikon Biostation microscope, a Nikon Inverted microscope or a Zeiss LSM 980 confocal microscope equipped with commercial software from the manufacturer (Nikon IM-Q v2.0, Zeiss ZEN Blue v3.1).
Data analysis	The data was analyzed using the following progamms: ImageJ (Fiji), containing the plugins StarDist (v0.9), MorphoLibJ (v1.6) and TrackMate (v7.1). Python 3 using cellpose (v2.3) and ilastik (v1.4). Matlab R2021b containing PIVlab (v3.08) (MathWorks). The analysis code was either open source or custom-made, as indicated in the Materials and Methods section. GraphPad Prism 9 (v9.5.9) was used for statistical computing and generation of graphs.<br>The simulation code is publicly available on GitHub using the following link: https://github.com/siavashmonfared/celadro_three_dimensional

For manuscripts utilizing custom algorithms or software that are central to the research but not yet described in published literature, software must be made available to editors and reviewers. We strongly encourage code deposition in a community repository (e.g. GitHub). See the Nature Portfolio guidelines for submitting code & software for further information.

Data

Policy information about availability of data

All manuscripts must include a data availability statement. This statement should provide the following information, where applicable:

- Accession codes, unique identifiers, or web links for publicly available datasets
- A description of any restrictions on data availability
- For clinical datasets or third party data, please ensure that the statement adheres to our policy

All data supporting the findings of this study are included within the paper and its Supplementary Information. Source data are provided for all graphs.

Research involving human participants, their data, or biological material

Policy information about studies with human participants or human data. See also policy information about sex, gender (identity/presentation), and sexual orientation and race, ethnicity and racism.

Reporting on sex and gender	Invasive primary breast tumours excised from female patients managed at Institut Curie – Hôpital René Huguenin (Saint-Cloud, France) have been analyzed.
Reporting on race, ethnicity, or other socially relevant groupings	n.a.
Population characteristics	All patients were treated at Institut Curie - Hopital Rene Huguenin (Saint-Coud, France). All patients (mean age 61 years, range 29-91 years) met the following criteria: primary unilateral nonmetastatic breast carcinoma with clinical, histological and biological data were available; no radiotherapy or chemotherapy before surgery; and full follow-up at Institut Curie - Hospital René Huguenin.
Recruitment	n.a.
Ethics oversight	The study was approved by the local ethics committee (Breast Group of René Huguenin Hospital, Saint-Cloud France).

Note that full information on the approval of the study protocol must also be provided in the manuscript.

Field-specific reporting

Please select the one below that is the best fit for your research. If you are not sure, read the appropriate sections before making your selection.

☒ Life sciences ☐ Behavioural & social sciences ☐ Ecological, evolutionary & environmental sciences

For a reference copy of the document with all sections, see nature.com/documents/nr-reporting-summary-flat.pdf

Life sciences study design

All studies must disclose on these points even when the disclosure is negative.

Sample size	The sample size was not pre-determined for any experiment. Sample sizes were chosen based on previous experience and publications in the same field (e.g. Saw et al., Nature, 2017; Balasubramaniam, Nature Materials, 2021; Sonam Nature Physics 2023).
Data exclusions	No data were excluded. Experiments with technical failures (e.g. loss of focus) were not analyzed.
Replication	We conducted at least two independent experimental replicates with several technical replicates for all experiments. The exact amount of experimental and technical replicates is specified in the text and the figure legends. All replicates were successful.
Randomization	No specific randomization method was used, samples were grouped depending on the specific experimental condition and treatment (e.g. comparing different substrate stiffnesses, drug treatment, etc.).
Blinding	No specific blinding was used. Our main conclusions are based on measuring forces and stresses, requiring computational processing after data acquisition. Thus, the acquisition is "blind", in a sense that the meaningful information is not accessible to the investigators at the time of data acquisition. Most analysis was done automatically, using computer programs, which does not require blinding.

Reporting for specific materials, systems and methods

We require information from authors about some types of materials, experimental systems and methods used in many studies. Here, indicate whether each material, system or method listed is relevant to your study. If you are not sure if a list item applies to your research, read the appropriate section before selecting a response.

Materials & experimental systems

n/a	Involved in the study
☐	☒ Antibodies
☐	☒ Eukaryotic cell lines
☒	☐ Palaeontology and archaeology
☒	☐ Animals and other organisms
☒	☐ Clinical data
☒	☐ Dual use research of concern
☒	☐ Plants

Methods

n/a	Involved in the study
☒	☐ ChIP-seq
☒	☐ Flow cytometry
☒	☐ MRI-based neuroimaging

Antibodies

Antibodies used

all primary antibodies were diluted 1:100
anti E-cadherin mouse antibody (catalog no 610181, BD Biosciences)
anti E-cadherin clone ECCD2 for 2D PDX staining catalog no 1319000 ThermoFisher)
anti alpha-catenin rabbit antibody (catalog no AB51032, Abcam)
anti beta-catenin rabbit antibody (catalog no 610156, BD Biosciences)
anti paxillin rabbit antibody (catalog no AB32084, Abcam)
anti phospho-myosin light chain 2 (pMLC2) rabbit antibody (catalog no 3671S, Cell Signaling)
anti ZO1 rabbit antibody (catalog no 402300, Life Technologies)
anti vimentin antibody (catalog no 8978, ThermoFisher)
anti phospho-Histone H3 mouse antibody (Ser 10, catalog no 9706, Cell Signaling)
anti desmoplakin mouse antibody (catalog no Cl.11-5F, Sigma)

all secondary antibodies were diluted 1:200
anti rabbit (catalog no A31573, Life Technologies)
anti mouse (catalog no A31571, Life Technologies)

Validation

All antibodies used are commercially available and were validated for research use by the manufacturers using functional testing of positive control samples.

Eukaryotic cell lines

Policy information about cell lines and Sex and Gender in Research

Cell line source(s)

Commercially available:
MDCK-II (ATCC CCL-34)
The following cell lines are not commercially available. They are described in the references and might be available upon request to the corresponding authors:
MDCK-II LifeAct-Ruby (Le et al., Nature Communications 2021)
MDCK-II E-cadherin knockout (Balasubramaniam et al., Nature Materials 2021)
MDCK-II E-cadherin knockout LifeAct-EGFP (Balasubramaniam et al., Nature Materials 2021)
MDCK-II E-cadherin/Cadherin 6 double knockout (Glentis et al., Science Advances 2022)
MDCK-II E-cadherin-GFP (Adams et al., Journal of Cell Biology 1998)
MCF10A EGFP (Villeneuve et al., PNAS 2019)
MCF10A E-cadherin knockout (Rhys et al., Journal of Cell Biology 2017)

Authentication

RNA-sequencing was performed for MDCK WT and E-cadherin knockout cells.

Mycoplasma contamination

Cells were regularly tested for mycoplasma contamination and found negative.

Commonly misidentified lines
(See ICLAC register)

No commonly misidentified cell lines were used.

Plants

Seed stocks

n.a.

Novel plant genotypes

n.a.

Authentication

n.a.
